# Expression of ROS-responsive genes and transcription factors after metabolic formation of H_2_O_2_ in chloroplasts

**DOI:** 10.3389/fpls.2012.00234

**Published:** 2012-11-01

**Authors:** Salma Balazadeh, Nils Jaspert, Muhammad Arif, Bernd Mueller-Roeber, Veronica G. Maurino

**Affiliations:** ^1^Institute of Biochemistry and Biology, University of PotsdamPotsdam, Germany; ^2^Plant Molecular Physiology and Biotechnology, Center of Excellence on Plant Sciences, Heinrich-Heine-UniversityDüsseldorf, Germany

**Keywords:** glycolate oxidase, H_2_O_2_, ROS-responsive genes, transcription factors

## Abstract

Glycolate oxidase (GO) catalyses the oxidation of glycolate to glyoxylate, thereby consuming O_2_ and producing H_2_O_2_. In this work, *Arabidopsis thaliana* plants expressing GO in the chloroplasts (GO plants) were used to assess the expressional behavior of reactive oxygen species (ROS)-responsive genes and transcription factors (TFs) after metabolic induction of H_2_O_2_ formation in chloroplasts. In this organelle, GO uses the glycolate derived from the oxygenase activity of RubisCO. Here, to identify genes responding to an abrupt production of H_2_O_2_ in chloroplasts we used quantitative real-time PCR (qRT-PCR) to test the expression of 187 ROS-responsive genes and 1880 TFs after transferring GO and wild-type (WT) plants grown at high CO_2_ levels to ambient CO_2_ concentration. Our data revealed coordinated expression changes of genes of specific functional networks 0.5 h after metabolic induction of H_2_O_2_ production in GO plants, including the induction of indole glucosinolate and camalexin biosynthesis genes. Comparative analysis using available microarray data suggests that signals for the induction of these genes through H_2_O_2_ may originate in the chloroplast. The TF profiling indicated an up-regulation in GO plants of a group of genes involved in the regulation of proanthocyanidin and anthocyanin biosynthesis. Moreover, the upregulation of expression of TF and TF-interacting proteins affecting development (e.g., cell division, stem branching, flowering time, flower development) would impact growth and reproductive capacity, resulting in altered development under conditions that promote the formation of H_2_O_2_.

## Introduction

Photosynthetic organisms are confronted with reactive oxygen species (ROS), such as singlet oxygen (^1^O_2_), the superoxide anion radical (O^−^_2_), the hydroxyl radical (OH·), and hydrogen peroxide (H_2_O_2_), which may cause oxidative stress and damage to important biological molecules (Apel and Hirt, 2004; Møller et al., 2007). Plants in their natural environments are often exposed to sudden increases in light intensity, which results in the absorption of excitation energy in excess of that required for metabolism. In chloroplasts, when absorbed energy is in excess at photosystem II (PSII), O^−^_2_ is produced during the Mehler reaction by Fd-NADPH oxidase at PSI and is dismutated by superoxide dismutase (SOD) to H_2_O_2_ (Ort and Baker, 2002; Asada, 2006). The photorespiratory pathway consumes photosynthetic reducing energy and produces H_2_O_2_ in the peroxisomes through the action of glycolate oxidase (GO) (Maurino and Peterhansel, 2010). H_2_O_2_ is also produced during a variety of different reactions under stress conditions, often through the detoxification of ^1^O_2_ and O^−^_2_. The generated H_2_O_2_ is scavenged by different antioxidant/enzyme reactions: the ascorbate and glutathione cycles, ascorbate peroxidase (APX), catalase, and peroxiredoxin (PRX) (Tripathi et al., 2009).

ROS generated in the chloroplast have been implicated as triggers of signaling pathways that influence expression of nuclear-encoded genes, which may initiate responses such as cell death or acclimation depending on the degree of the stress (Karpinski et al., 1999; Fryer et al., 2003; Op den Camp et al., 2003; Danon et al., 2005). H_2_O_2_ can take part in signaling acting as messenger either directly (e.g., by reversibly modifying critical thiol groups in target proteins; Neill et al., 2002) or by using an oxidized product as a secondary messenger (Møller et al., 2007). The H_2_O_2_-scavenging enzymes APX and dehydroascorbate reductase (DHAR) may act as highly efficient initiators of oxidative signaling by generating transient bursts of reduced glutathione. This in consequence triggers glutaredoxin-mediated protein oxidation (Neill et al., 2002). Crosstalk between redox pools of different cellular compartments, possibly transmitted by a redox shift in cellular components, has also been suggested to be important for control of the expression of nuclear genes (Baier and Dietz, 2005; Leister, 2005). A generalized model of H_2_O_2_ signal transduction pathways suggests that H_2_O_2_ may also directly oxidize transcription factors (TFs) in either the cytosol or the nucleus. Alternatively, H_2_O_2_-mediated activation of a signaling protein such as a protein kinase may activate TFs (Mittler et al., 2004; Miao et al., 2007). TFs would interact with cognate H_2_O_2_-response elements in target gene promoters thereby modulating gene expression (Foyer and Noctor, 2005). Recently, Møller and Sweetlove (2010) put forward the hypothesis that H_2_O_2_ itself is unlikely to be the signaling molecule that selectively regulates nuclear-encoded chloroplastic genes but rather that oxidized peptides deriving from proteolysis of oxidized proteins would act as second messengers during retrograde ROS signaling. On the other hand, using spin trapping EPR spectroscopy in addition to chemical assays (employing Amplex Red reagent), Mubarakshina et al. (2010) showed that 5% of the H_2_O_2_ produced inside chloroplasts at high light intensities can actually be detected outside the organelles. This process may involve the pass of H_2_O_2_ through aquaporins (Bienert et al., 2007) and might be sufficient to trigger signaling processes outside the chloroplasts.

Desikan et al. (2001) showed that approximately 1% of the transcriptome was altered in H_2_O_2_-treated *Arabidopsis thaliana* (*A. thaliana*) cell cultures. Although H_2_O_2_-responsive promoters have been identified (Desikan et al., 2001), specific H_2_O_2_-regulatory DNA sequences and their cognate TFs have not been isolated and characterized. In more recent studies genes involved in H_2_O_2_ signal transduction have been identified or proposed, including mitogen-activated protein kinases (MAPKs), various TFs of e.g., the NAC, ZAT, and WRKY families, miRNAs and others (Van Breusegem et al., 2008; Li et al., 2011; Petrov and Van Breusegem, 2012). Moreover, using genome-wide analysis of catalase deficient *A. thaliana*, H_2_O_2_ was inferred to regulate the expression of genes encoding specific small heat shock proteins, several TFs and candidate regulatory proteins (Vandenabeele et al., 2004; Vanderauwera et al., 2005).

To date, it is not known to which extent the chemical specificity of the ROS and the cellular compartment of their release may contribute to the multiplicity of responses that occur in plants. A major challenge is to dissect the genetic networks that control ROS signaling and to assess specific and common responses toward different types of ROS signals. To this end, the molecular, biochemical and physiological responses of *A. thaliana* to elevated *in planta* levels of H_2_O_2_ were and are being investigated in various types of model systems including mutants altered in the ROS scavenging machinery (Maurino and Flügge, 2008). However, the analysis of dynamic physiological processes using (knock-out) mutants may not always be straightforward, especially when compensatory cellular mechanisms are induced. With respect to ROS-related mutants, changing the balance of scavenging enzymes may induce compensatory mechanisms such that signaling and oxidative damage effects may not be easily separated. Moreover, invasive experimental setups like the application of oxidative stress-causing agents may induce a non-specific oxidative stress that acts throughout the cell and triggers additional responses that may complicate the analysis of ROS signal transduction pathways (Maurino and Flügge, 2008). We have recently developed a tool to functionally dissect the action of plastid-generated H_2_O_2_, using plants overexpressing GO in plastids (GO plants; Fahnenstich et al., 2008). During photosynthesis, the oxygenase activity of ribulose 1,5-bisphosphate carboxylase/oxygenase (RubisCO) produces glycolate 2-phosphate within the chloroplasts, which is then dephosphorylated to glycolate by phosphoglycolate phosphatase (Maurino and Peterhansel, 2010). In GO plants, glycolate is oxidized to glyoxylate by the plastidic GO, with the parallel production of H_2_O_2_. When growing under moderate photon fluxes and ambient CO_2_ concentration (photorespiratory conditions) the GO plants remain smaller than the wild type, presenting a reduced rosette diameter and yellowish leaves due to H_2_O_2_ accumulation (Fahnenstich et al., 2008). In contrast, in non-photorespiratory conditions (e.g., at high CO_2_ concentration) the oxygenase activity of RubisCO is abolished and thus, the metabolic flux through GO is suppressed, allowing GO plants to grow like wild type (Fahnenstich et al., 2008). Transferring GO plants from high to ambient CO_2_ concentration specifically induces H_2_O_2_ formation in the chloroplasts (Fahnenstich et al., 2008). These properties permit the modulation of plastidic produced H_2_O_2_ levels by changing light intensity and/or CO_2_ levels (Maurino and Flügge, 2008). Moreover, H_2_O_2_ is specifically generated without a concomitant accumulation of superoxide or singlet oxygen, which are common precursors of H_2_O_2_ during ROS generation in chloroplasts. A similar experimental set-up was employed in previous studies using catalase null mutants in which the production of peroxisomal H_2_O_2_ is induced by changing the conditions of plant growth from non-photorespiratory to photorespiratory conditions (e.g., high light intensity) (Dat et al., 2000; Vandenabeele et al., 2004; Vanderauwera et al., 2005). The metabolic production of H_2_O_2_ may avoid the pleiotropic effects discussed above but it cannot be ruled out that ROS-unrelated pleiotropic reactions may occur in both approaches due to abrupt changes in CO_2_ level or light intensity.

In this work we attempted to identify genes strongly responding to an abrupt production of H_2_O_2_ in chloroplasts of *A. thaliana*. To this end we tested the expressional changes of 187 nuclear-encoded ROS-responsive genes and 1880 TFs, using quantitative real-time (qRT)-PCR (Czechowski et al., 2004; Balazadeh et al., 2008; Wu et al., 2012) upon transfer of high CO_2_-grown GO and wild-type (WT) plants to ambient CO_2_ concentration. Our data revealed a rapid and coordinated expression response of ROS-affected genes of specific functional networks in GO including an early induction of indole glucosinolate and camalexin biosynthesis genes and an up-regulation of a group of genes involved in the regulation of proanthocyanidin and anthocyanin biosynthesis. Moreover, the upregulation of expression of TF and TF-interacting proteins affecting development (e.g., cell division, stem branching, flowering time, flower development) would impact growth and reproductive capacity, resulting in altered development under conditions that promote the formation of H_2_O_2_.

## Materials and methods

### Plant material

*Arabidopsis thaliana* (L.) Heynh. ecotype Columbia-0 (Col-0, wild-type) constitutively expressing glycolate oxidase (GO, At3g14420) in the plastids (GO plants) under the cauliflower mosaic virus 35S promoter were generated in our previous work (Fahnenstich et al., 2008). In these plants to direct the expression of GO to the chloroplats the stromal targeting presequence from *Arabidopsis thaliana* phosphoglucomutase (At5g51820) was used (Fahnenstich et al., 2008). WT and GO transgenic plants were grown in pots containing 3 parts of soil (Gebr. Patzer KG, Sinntal-Jossa, Germany) and one part of vermiculite (Basalt Feuerfest, Linz, Austria) under a 16 h-light/8 h-dark regime at photosynthetically active photon flux densities (PPFD) of 75 μmol quanta m^−2^ s^−1^ at 22°C day/18°C night temperatures and a CO_2_ concentration of 3000 ppm. After 3 weeks of growth plants were transferred to ambient CO_2_ concentration (380 ppm) and the same PPFD. Whole rosettes were harvested at different time points after transfer, immediately frozen in liquid nitrogen and stored at −80°C until use for RNA isolation and H_2_O_2_ measurements.

### Isolation of RNA and real-time PCR analysis

For the large-scale qRT-PCR analysis, total RNA was extracted from 100 mg leaves (fresh weight) using RNeasy Plant Mini kit (Qiagen, Valencia, USA) according to the manufacturer's protocol. DNAse I digestion was performed on 20–30 μg of total RNA using TURBO DNase Kit (Ambion, Cambridgeshire, UK) according to manufacturer's instructions. RNA integrity was checked on 1% (w/v) agarose gels and concentration measured with a Nanodrop ND-1000 spectrophotometer before and after DNAse treatment. Absence of genomic DNA was confirmed subsequently by quantitative PCR using primers that amplify an intron sequence of the gene At5g65080 (forward 5′-TTTTTTGCCCCCTTCGAATC-3′ and reverse 5′-ATCTTCCGCCACCACATTGTAC-3′). First-strand cDNA was synthesized from 8 μg to 10 μg of total RNA using RevertAid™ First Strand cDNA Synthesis Kit (Fermentas, St. Leon-Rot, Germany) following the manufacturer's protocol. The efficiency of cDNA synthesis was estimated by qRT-PCR using two different primer sets annealing to the 5′ and 3′ ends, respectively, of a control gene (At3g26650, *GAPDH*, glyceraldehyde-3-phosphate dehydrogenase). Primer sequences were as follows: for GAPDH3′, forward 5′-TTGGTGACAACAGGTCAAGCA-3′ and reverse 5′-AAACTTGTCGCTCAATGCAATC-3′; for GAPDH5′, forward 5′-TCTCGATCTCAATTTCGCAAAA-3′ and reverse 5′-CGAAACCGTTGATTCCGATTC-3′. Transcript levels of each gene were normalized to *ACTIN2* (At3g18780) transcript abundance (forward 5′-TCCCTCAGCACATTCCAGCAGAT-3′ and reverse 5′-AACGATTCCTGGACCTGCCTCATC-3′). A total of 187 ROS-responsive genes (Wu et al., 2012) and 1880 TFs (Czechowski et al., 2004; Balazadeh et al., 2008) were analyzed by qRT-PCR as previously described (Caldana et al., 2007; Balazadeh et al., 2008). PCR reactions were run on an ABI PRISM 7900HT sequence detection system (Applied Biosystems, Darmstadt, Germany), and amplification products were visualized using SYBR Green (Applied Biosystems).

### H_2_O_2_ measurements

Levels of H_2_O_2_ were determined using the Amplex® Red Technology (Life Technologies, Darmstadt, Germany) following the manufacturer's instructions. Amplex Red (N-acetyl-3,7-dihydroxyphenoxazine) reacts with H_2_O_2_ in the presence of horseradish peroxidase and forms the fluorescent product resorufin. For the determinations, 100 mg leaves (fresh weight) were ground in liquid nitrogen into a fine powder and resuspended with 0.15 mL extraction buffer prepared as indicated by the manufacturer. This suspension was centrifuged at 4°C at 13,000 rpm for 15 min. Five μL of the supernatant, 45 μL distilled water and 50 μL of Amplex® Red solution were added to a microtitre plate. After 30 min incubation in the dark fluorescence was measured by excitation at 560 nm and emission reads at 590 nm. A calibration curve was established with known H_2_O_2_ concentrations.

### Gene expression network analysis

The two genes that were most strongly induced under photorespiratory conditions in GO plants at the 0.5 and 6 h time points (At3g02840 and At1g17180, respectively) were used as baits to identify globally coexpressed genes using the ATTED-II database (http://atted.jp), which allows evaluating genes that are coexpressed under five experimental conditions (tissue, abiotic stress, biotic stress, hormones, and light conditions) (Obayashi et al., 2009).

## Results and discussion

### Induction of H_2_O_2_ formation in GO plants

The production of H_2_O_2_ in leaves of plants overexpressing GO in the plastids (Fahnenstich et al., 2008) was analyzed after activation of photorespiration by transferring high CO_2_-grown plants to ambient-CO_2_ conditions. As shown in Table 1, higher levels of H_2_O_2_ were determined in GO than in WT plants at 0.5 and 4 h after transfer while GO and WT plants maintained under non-photorespiratory conditions (3000 ppm CO_2_) showed similar H_2_O_2_ levels at both time points (Table 1). Note, that as the measurements were performed using whole-leaf extracts the expected differences in chloroplastic H_2_O_2_ levels between GO and WT plants under photorespiratory condition may be higher than determined here.

**Table 1:** **Levels of H_2_O_2_ measured in whole rosettes (μmol/g FW) after shifting high CO_2_-grown wild-type and GO plants to ambient CO_2_ concentration for 0.5 and 4 h**.

	**0.5 h**		**4 h**	
	**High CO**_**2**_	**Ambient CO**_**2**_	**High CO**_**2**_	**Ambient CO**_**2**_
WT	2.4 ± 0.2	2.3 ± 0.2	2.5 ± 0.4	2.7 ± 0.1
GO	2.5 ± 0.3	3.0 ± 0.3	2.6 ± 0.2	**3.4** ± **0.0**

Samples from control plants maintained in high CO_2_ were processed in parallel. Values indicate the mean ± SE of three independent samples and those set in bold face indicate significant differences to the corresponding wild-type value calculated by Student's t-test (P < 0.05). WT, wild type.

### Expression profiling of ROS marker genes in GO and wild-type plants after the induction of H_2_O_2_ formation in chloroplasts

To study the impact of an abrupt production of H_2_O_2_ in chloroplasts on nuclear gene expression, we analyzed transcript level changes of 187 ROS-responsive genes using a previously established qRT-PCR platform (detailed in Wu et al., 2012). The genes included in the platform were chosen from published reports and our own experiments and represent four different groups that were already shown to be rapidly induced by (1) superoxide radical (O^−^_2_; 18 genes), (2) singlet oxygen (^1^O_2_; 22 genes), (3) H_2_O_2_ (53 genes), or (4) different types of ROS (general ROS-responsive genes; 94 in total).

Gene expression was analyzed in whole rosettes of 3-week-old WT and GO plants at 0.5, 4, 6, and 12 h after shifting high-CO_2_-grown plants (non-photorespiratory condition) to ambient CO_2_ concentration (photorespiratory condition). Expression profiling was performed in two biological replicates and log-fold change (log2 FC) ratios of expression changes were calculated for GO and WT plants by comparing gene expression levels before and after the CO_2_ concentration shift. A total of 131 genes were expressed in all examined samples (Table A1 in Appendix). The remaining 56 genes did not yield detectable PCR amplicons, indicating no or marginal expression under our experimental conditions.

**Figure 1:**
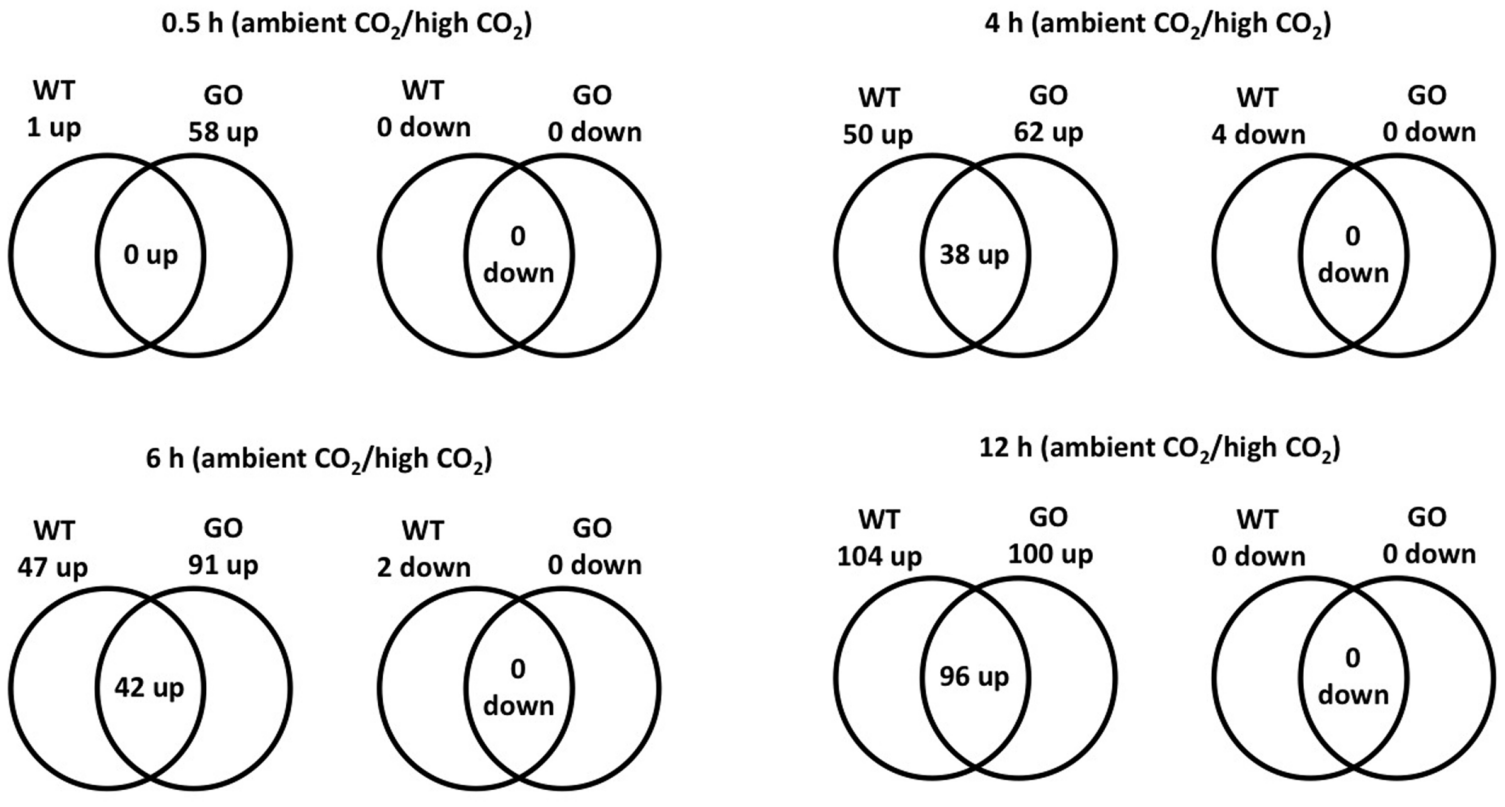
**Venn diagram of the number of ROS-responsive genes differentially expressed in wild-type and GO plants at different time points (0.5, 4, 6, and 12 h) after the transfer of plants grown at high CO_2_ concentration (3000 ppm) to ambient CO_2_ concentration (380 ppm)**.

Considering a 3-fold expression difference cut-off, 120 genes displayed differential expression in GO and/or WT plants upon transfer from high to ambient CO_2_ concentration; the vast majority of the affected genes (116 in total) were up-regulated, and only four genes were down-regulated (Figure 1, Table A1 in Appendix). Most noticeably, expression of 58 genes was induced in GO plants already within 0.5 h after the transfer to ambient CO_2_ condition, whilst only a single gene was induced in the wild type at the same time point (Figure 1). Importantly, however, many genes showed also high expression in the wild type at later time points after the CO_2_ concentration shift, but the expressional changes were in most cases more pronounced in GO than WT plants (Figure 1, and section “Early Induction of Indole Glucosinolate and Camalexin Biosynthesis Genes in GO Plants”). Thus, our data indicate that similar sets of ROS-responsive genes responded to the CO_2_ shift in GO and WT plants; however, the dynamics of the transcriptional responses were clearly different in the two types of plants, being faster and more prominent in the GO plants.

### Early induction of indole glucosinolate and camalexin biosynthesis genes in GO plants

To identify transcripts responsive to metabolically produced H_2_O_2_ we focused our analysis on the 0.5- and 6-h time points. Genes were considered differentially expressed when the fold change was more than 3-fold (log_2_ ≥ 1.56).

At 0.5 h after shifting plants to ambient CO_2_ concentration, 58 of the 131 expressed genes were induced in GO plants by more than 3-fold, whilst in the wild type the expression change was less than 3-fold, suggesting that these genes participate in early signaling steps triggered by the production of H_2_O_2_ under photorespiratory conditions (Table 2). After 6 h, seven of these genes showed WT levels of expression (below 3-fold), while 29 were further overexpressed only in GO (Table 2). Although at 6 h after transfer to ambient CO_2_ the expression fold-change (FC) of the remaining 22 genes was higher than 3 in both, GO and WT plants, the expression change between GO and WT (FC_GO_/FC_WT_) was higher than 2 for 16 of these genes (Table 2), indicating that their higher expression in GO plants was triggered by the elevated levels of H_2_O_2_.

**Table 2:** **ROS-responsive genes (58 in total) the expression of which was enhanced by more than 3-fold in GO plants 0.5 h after shifting plants grown at high CO_2_ concentration (3000 ppm) to ambient CO_2_ concentration (380 ppm)**.

**AGI**	**0.5 h**			**6 h**			**Annotation**
	**FC**_**WT**_	**FC**_**GO**_	**FC**_**GO**_**/FC**_**WT**_	**FC**_**WT**_	**FC**_**GO**_	**FC**_**GO**_**/FC**_**WT**_	
**UP-REGULATED IN GO AT 0.5 H**							
**At1g69890**	1.3	15.9	**12.0**	1.2	2.2	1.8	Protein of unknown function
At2g40000^*^	0.9	10.2	**11.2**	1.6	2.1	1.3	Ortholog of sugar beet HS1 PRO-1 2 (HSPRO2)
At2g18210	1.0	8.4	**8.2**	1.1	2.9	**2.6**	Protein of unknown function
**At1g18570**	1.2	6.8	**5.7**	0.7	2.5	**3.6**	Myb-type transcription factor (HIG1/MYB51)
At1g21100	1.6	6.9	**4.2**	0.7	1.5	**2.2**	Indole glucosinolate O-methyltransferase (IGMT1)
**At5g64310**	1.2	3.8	**3.1**	3.1	1.1	0.4	Arabinogalactan protein (AGP1C) of unknown function
**At5g28630**	1.4	4.0	**2.9**	0.3	0.4	1.5	Protein of unknown function
**UP-REGULATED IN GO AT 0.5 H AND 6 H**							
**CHANGE IN GENE EXPRESSION IN WT AT 6 H < 3**							
At3g02840^*^	1.1	79.3	**71.2**	2.9	65.4	**22.8**	Putative U-box-type E3 ubiquitin ligase
At2g37430^*^	0.9	53.3	**62.7**	1.9	190.8	**100.9**	C2H2 and C2HC zinc fingers superfamily protein (ZAT11)
At1g05575^*^	1.5	45.6	**29.9**	0.6	4.3	**6.8**	Protein of unknown function
**At2g38470**	1.4	31.5	**22.3**	1.8	7.5	**4.3**	WRKY-type transcription factor (WRKY33)
**At4g17490**	1.5	21.5	**14.7**	1.3	6.9	**5.2**	Ethylene-responsive element binding factor (ERF6)
**At5g47230**	1.3	17.4	**13.8**	1.5	4.4	**3.0**	Ethylene-responsive element binding factor (ERF5)
At1g66060	1.3	17.5	**13.2**	2.7	5.1	1.9	Protein of unknown function
**At2g32030**	1.3	16.4	**12.2**	1.9	24.5	**12.9**	Putative GNAT-type N-acetyltransferase
At2g26530^*^	1.2	13.3	**10.7**	2.1	5.4	**2.5**	Protein of unknown function; AR781
**At1g21120**	1.0	9.9	**10.3**	0.7	15.9	**22.1**	Indole glucosinolate O-methyltransferase (IGMT2)
**At1g35210**	1.1	11.2	**10.2**	2.9	14.4	**4.9**	Protein of unknown function
At3g55980^*^	1.4	14.3	**10.1**	1.2	4.0	**3.3**	Salt-inducible zinc finger 1, SZF1 (C3H47)
At2g33710	1.0	7.5	**7.7**	0.8	12.6	**15.0**	Putative ERF-type transcription factor
At2g25735^*^	1.8	10.8	**5.9**	0.6	3.2	**5.7**	Protein of unknown function
**At5g54490**	1.5	7.8	**5.3**	1.5	4.1	**2.8**	PBP1, Pinoid Binding Protein 1
At1g19020^*^	1.8	9.3	**5.1**	1.9	27.7	**14.5**	Protein of unknown function
**At5g51190**	1.4	6.8	**4.9**	1.8	5.0	**2.7**	Putative ERF-type transcription factor
At3g02800^*^	1.1	5.0	**4.5**	1.2	4.1	**3.4**	Tyrosine phosphatase (ATPFA-DSP3)
**At5g64905**	1.4	6.0	**4.4**	2.1	33.0	**16.0**	Putative peptide elicitor Pep3 precursor protein (ProPep3)
At1g76600^*^	1.4	5.8	**4.3**	1.7	11.0	**6.3**	Protein of unknown function
**At3g23230**	2.2	9.4	**4.2**	2.3	17.1	**7.5**	Putative ERF-type transcription factor (ERF98)
At1g59590	1.3	5.2	**4.1**	1.4	5.2	**3.8**	Zinc finger protein (ZCF37) of unknown function
At4g18880^*^	1.6	6.5	**4.0**	1.9	4.6	**2.4**	Heat stress-type transcription factor (HsfA4a/HSF21)
**At2g41640**	1.1	4.0	**3.7**	2.2	6.0	**2.7**	Protein of unknown function
At1g28190^*^	1.1	3.9	**3.5**	2.4	5.6	**2.3**	Protein of unknown function
**At5g57220**	2.8	9.1	**3.3**	1.3	6.9	**5.2**	Cytochrome P450 monooxygenase (CYP81F2)
At1g26380^*^	1.2	3.4	**3.0**	2.1	39.0	**18.9**	UDP-N-acetylmuramate dehydrogenase of unknown function
At2g31945	1.3	3.5	**2.7**	2.3	5.1	**2.2**	Protein of unknown function
**At4g11280**	1.5	3.0	**2.0**	1.4	5.9	**4.3**	1-Aminocyclopropane-1-carboxylate synthase (ACS6)
**CHANGE IN GENE EXPRESSION IN WT AT 6 H > 3**							
**At1g80840**	1.1	38.0	**35.4**	5.5	56.4	**10.3**	WRKY-type transcription factor (WRKY40)
At5g04340^*^	0.8	27.5	**32.6**	7.3	8.6	**1.2**	C2H2-zinc-finger-TF (C1-2iD-04) of unknown function
**At2g22880**	1.7	50.8	**29.4**	6.2	28.1	**4.5**	Protein of unknown function
**At1g27730**	1.1	30.1	**26.8**	6.0	28.0	**4.7**	C2H2-zinc-finger-TF (ZAT10/STZ)
**At5g27420**	1.2	32.7	**26.3**	3.0	15.0	**5.0**	Putative ubiquitin ligase, ATL subfamily (ATL31)
**At1g61340**	1.5	24.3	**16.3**	6.6	10.5	1.6	ATFBS1. F-Box stress induced 1 of unknown function
**At5g59820**	1.0	14.3	**14.1**	4.6	61.7	**13.5**	C2H2-zinc-finger-TF (ZAT12)
At5g24110^*^	1.0	13.3	**13.8**	3.3	91.2	**28.0**	WRKY-type transcription factor (WRKY30)
**At4g24570**	1.4	18.8	**13.6**	3.6	3.3	0.9	Dicarboxylate carrier (DIC2)
**At3g10930**	1.7	19.0	**11.5**	8.5	38.8	**4.6**	Protein of unknown function
At3g25250^*^	1.5	17.3	**11.4**	11.1	153.6	**13.8**	Putative protein kinase (AGC2/OXI1)
At4g39670^*^	1.3	14.3	**10.7**	31.7	184.9	**5.8**	Sphingosine transfer protein; accelerated death 11 (ACD11)
At1g77450^*^	0.8	7.3	**9.5**	5.6	6.9	1.2	NAC-type transcription factor (ANAC032)
**At1g72520**	1.7	15.7	**9.3**	4.8	9.7	**2.0**	Lipoxygenase (LOX4)
**At3g48650**	1.9	14.8	**7.6**	6.2	7.4	1.2	14a-related protein of unknown function
**At4g21390**	1.8	13.0	**7.0**	6.5	28.9	**4.4**	Putative S-domain-type receptor protein kinase
At5g63790^*^	0.8	5.4	**7.0**	3.1	11.1	**3.6**	NAC-type transcription factor (ANAC102)
At4g37370	1.4	7.0	**5.0**	2.8	67.5	**24.1**	Cytochrome P450 monooxygenase (CYP81D8)
At1g63720	1.5	5.0	**3.2**	3.1	6.1	1.9	Hydroxyproline-rich glycoprotein family protein
At2g18690	1.5	4.4	**3.0**	6.6	24.7	**3.7**	Protein of unknown function
At1g05340^*^	1.2	3.4	**2.7**	4.3	7.9	1.8	Protein of unknown function
**At1g57630**	1.4	3.4	**2.4**	20.2	36.0	1.8	Protein of unknown function

Genes are listed according to the difference of the expression change between GO and wild-type (WT) plants (FC_GO_/FC_WT_) at 0.5 h. FC_GO_/FC_WT_ values higher than 2 are shown in bold face. AGI: gene identification number given by the Arabidopsis Genome Initiative. Genes also induced in catalase loss-of function mutants are highlighted with an asterisk (

* ) (Inzé et al., 2012). Genes included in the same gene coexpression network of At3g02840 (putative U-box-type E3 ubiquitin ligase) are highlighted in bold face (http://atted.jp; Obayashi et al., 2009). The gene annotation was retrieved from TAIR (http://arabidopsis.org/index.jsp).

Later responding genes, which were affected only after 6 h under photorespiratory conditions, were also identified. From the 23 genes that showed an expression change of above 3-fold in GO, 13 were only induced in GO, while 10 genes were induced in both, GO and WT. The FC ratio in GO and WT (FC_GO_/FC_WT_) was above 2 for the 10 genes (Table 3), indicating that their expression in GO plants is controlled by the higher levels of H_2_O_2_, similar to the early-responsive genes.

**Table 3:** **ROS-responsive genes (23 in total) the expression of which was enhanced more than 3-fold in GO plants 6 h after shifting plants grown at high CO_2_ concentration to ambient CO_2_ concentration**.

**AGI**	**0.5 h**			**6 h**			**Annotation**
	**FC**_**WT**_	**FC**_**GO**_	**FC**_**GO**_**/FC**_**WT**_	**FC**_**WT**_	**FC**_**GO**_	**FC**_**GO**_**/FC**_**WT**_	
**UP-REGULATED IN GO AT 6 H**							
**CHANGE IN GENE EXPRESSION IN WT < 3**							
At1g26420	1.5	1.6	1.1	2.4	17.6	**7.3**	Putative reticuline dehydrogenase
**At2g15480**	1.0	2.8	**2.8**	1.2	7.2	**6.0**	UDP-dependent glycosyl transferase (UGT73B5)
At1g10040	1.2	1.8	1.5	2.1	10.4	**5.0**	Putative hydrolase
**At2g29490**	0.7	1.9	**2.8**	2.3	10.4	**4.5**	Tau glutathione S-transferase (GSTU1)
At5g46080	1.1	1.9	1.8	1.2	3.7	**3.1**	Putative protein kinase
At1g80820	1.2	1.5	1.3	2.5	7.8	**3.1**	Cinnamoyl CoA reductase, involved in lignin biosynthesis
At3g09410	1.2	0.8	0.7	1.2	3.2	**2.7**	Putative pectin acetylesterase
At2g29500^*^	1.0	1.1	1.2	1.5	3.8	**2.5**	HSP20-type protein (HSP17.6B-CI); unknown function
At4g22530^*^	1.3	0.8	0.7	2.4	5.9	**2.4**	Putative methyltransferase
At4g15975	1.7	1.3	0.7	1.6	3.7	**2.4**	Putative ubiquitin ligase (RRE4/ATL17)
At2g38340	1.0	0.7	0.7	2.7	6.1	**2.2**	Putative AP2-type transcription factor (DREB2E)
At3g13790	1.3	1.3	1.1	2.5	5.0	**2.0**	Putative cell wall invertase (CwINV1)
At1g76070	1.2	2.6	**2.2**	1.6	3.3	**2.0**	Protein of unknown function
**CHANGE IN GENE EXPRESSION IN WT > 3**							
**At1g17180**	0.6	0.9	1.4	7.5	104.0	**13.8**	Tau glutathione S-transferase (GSTU25)
**At1g15520**	1.2	0.8	0.7	12.4	111.9	**9.0**	ABC transporter (ABCG40/PDR12)
**At1g17170**	1.0	1.3	1.4	5.7	40.4	**7.0**	Tau glutathione S-transferase (GSTU24)
At1g74360	1.0	2.2	**2.2**	4.2	14.2	**3.4**	Putative LRR-type receptor protein kinase
At2g38250^*^	1.2	1.7	1.4	4.4	13.7	**3.1**	Putative trihelix-type transcription factor
**At5g51060**	1.3	1.0	0.7	14.4	44.0	**3.1**	Respiratory burst oxidase homolog (AtRBOHC/RHD2)
At5g20230	1.5	2.8	1.8	9.9	28.3	**2.9**	Senescence associated gene (BCB/SAG14)
**At2g41380**	1.1	1.2	1.1	9.6	21.2	**2.2**	Putative S-adenosyl-L-methionine-dependent methyltransferase
At1g13340	1.0	2.1	**2.0**	3.4	6.8	**2.0**	Protein of unknown function
At5g48850	1.1	0.8	0.7	3.3	7.4	**2.2**	Protein of unknown function (ATSDI1)

Genes are listed according to the difference of the expression change between GO and wild-type (WT) plants (FC_GO_/FC_WT_) at 6 h. FC_GO_/FC_WT_ values higher than 2 are shown in bold face. AGI: gene identification number given by the Arabidopsis Genome Initiative. Genes also induced in catalase loss-of function mutants are highlighted with an asterisk (

* ) (Inzé et al., 2012). Genes included in the same gene coexpression network of At1g17180 (GSTU25) are highlighted in bold face (http://atted.jp; Obayashi et al., 2009). The gene annotation was retrieved from TAIR (http://arabidopsis.org/index.jsp).

The most highly up-regulated gene in GO plants at 0.5 h after induction of H_2_O_2_ production was At3g02840 (encoding a putative U-box-type E3 ubiquitin ligase, known to respond immediately-early to fungal elicitation) (Table 2). We used the ATTED-II database (http://atted.jp; Obayashi et al., 2009) to discover genes coexpressed with At3g02840 and observed that 45 of the 58 genes induced at 0.5 h after induction of H_2_O_2_ production cluster together (Table 2), indicating that metabolically produced H_2_O_2_ in GO plants induces the coordinate expression of functionally related genes. A similar analysis using the most highly expressed gene at 6 h after induction of H_2_O_2_ production (At1g17180, encoding glutathione S-transferase Tau 25) indicated that another group of eight genes are coordinately expressed in GO plants at this later time point (Table 3).

Recently, Inzé et al. (2012) listed the 85 most strongly H_2_O_2_-responsive genes in catalase loss-of-function mutants shifted from low- to high-light conditions, where H_2_O_2_ is produced in peroxisomes by the action of photorespiratory GOs. Interestingly, 23 of the 81 genes, which changed their expression in the GO plants were also differentially expressed in catalase loss-of-function mutants (Tables 2 and 3), indicating that they respond to enhanced levels of H_2_O_2_ independent of the site of its generation; the remaining genes may then represent candidates preferentially responsive to H_2_O_2_ produced in chloroplasts. Many of the genes up-regulated in GO plants encode proteins or TFs of currently unknown specific functions. Interestingly, however, several of the early-responsive genes are involved in tryptophan-derived biosynthesis of the phytoanticipins camalexin and indole glucosinolates, i.e., secondary metabolites that have antifungal and insect-deterring functions (Kliebenstein et al., 2001; Bednarek et al., 2009). These genes encode (1) the transcription factor WRKY33 (At2g38470), which is involved in controlling camalexin biosynthesis (Birkenbihl et al., 2012); (2) the Myb-type transcription factor HIG1/MYB51 (At1g18570) involved in the positive regulation of indole glucosinolate biosynthesis by activating several target genes (Gigolashvili et al., 2007); (3) the O-methyltransferases IGMT1 (At1g21100) and IGMT2 (At1g21120), which catalyze the transfer of a methyl group to the hydroxy indole glucosinolate hydroxyindol-3-ylmethylglucosinolate (4 and 1OH-I3M, respectively) to form methoxyindol-3-ylmethylglucosinolate (4 and 1MO-I3M, respectively) (Pfalz et al., 2011); and (4) cytochrome P450 monooxygenase CYP81F2 (At5g57220), that is essential for the pathogen-induced accumulation of 4-methoxyindol-3-ylmethylglucosinolate (4MI3G) (Bednarek et al., 2009). Our data thus show the early induction of indole glucosinolate and camalexin biosynthesis genes in GO plants after metabolic formation of H_2_O_2_ through the activation of genes encoding enzymes involved in intermediate metabolite conversions and of TFs that act on several target genes of these biosynthetic pathways.

### Transcription factor profiling

To understand the potential effects of overexpression of GO in chloroplasts on the nuclear transcriptional program, we next broadened our analysis by testing the expression of 1880 TFs using a highly sensitive quantitative real-time PCR (qRT-PCR) platform (Czechowski et al., 2004; Balazadeh et al., 2008). Considering the data obtained from the profiling of the ROS-responsive genes, we analyzed the expression at 0.5 h after induction of H_2_O_2_ production to capture the early-responsive TFs. Expression profiling was performed in two biological replicates and log-fold change (log2 FC) ratios of expression changes were calculated for GO and WT plants by comparing gene expression levels before and after the transfer of plants grown at high CO_2_ to ambient CO_2_.

TFs most strongly responding to H_2_O_2_ were identified by comparing their expression FC in GO and WT plants. A TF was considered differentially expressed when the FC in GO was more than 3-fold (log_2_ ≥ 1.56) and less than 2-fold in the wild type (log_2_ ≥ 1.0) (Table 4). Analysis of transcript profiles revealed that the expression of 1449 genes, representing 77% of all TF genes tested, could be detected (Table A2 in Appendix). The remaining 23% (431 of the 1880 TFs) did not yield detectable PCR amplicons, indicating no or very weak expression in the tested material.

**Table 4:** **Transcription factors the expression of which was enhanced by more than 3-fold in GO plants, but less than 2-fold in wild-type plants 0.5 h after shifting plants grown at high CO_2_ concentration to ambient CO_2_ concentration**.

**AGI**	**0.5 h after transfer to ambient CO**_**2**_			**Gene family**	**Annotation**	**Function**	**FG**
	**FC**_**WT**_	**FC**_**GO**_	**FC**_**GO**_**/FC**_**WT**_				
At5g19790	0.2	26.9	176.5	AP2/EREBP	RAP2.11	Modulates response to low potassium	4
At5g56200	0.1	14.5	169.0	C2H2	C1-4iB-01	Unknown function	5
At5g32460	1.3	123.7	92.9	B3	B3	Unknown function	5
At4g09820	0.8	34.4	45.5	bHLH	TT8	Regulation of proanthocyanidin and anthocyanin biosynthesis; affects dihydroflavonol 4-reductase gene expression.	1
At2g37430	1.9	80.4	43.3	C2H2	ZAT11	Unknown function	5
At1g48150	0.1	3.6	38.9	MADS	AGL74	Unknown function	5
At2g34600	0.4	8.4	24.1	ZIM	JAZ7	Jasmonate signaling; cambium regulator	3
At3g07260	0.8	19.0	22.7	FHA		Unknown function	5
At1g66380	1.9	40.0	21.6	MYB	MYB114	Regulates later steps of anthocyanin biosynthesis	1
At1g27730	1.8	36.3	20.5	C2H2	ZAT10/STZ	Involved in plant defense responses	4
At1g56650	0.6	12.1	20.1	MYB	MYB75	Involved in anthocyanin metabolism; regulates dihydroflavonol reductase expression	1
At5g37415	0.5	8.8	17.6	MADS	AGL105	Unknown function	5
At3g53340	0.4	6.5	17.5	CCAAT-HAP3	NF-YB10	Unknown function	5
At4g00250	0.4	6.3	16.8	GeBP	–	Indirect regulation of cytokinin response genes	2
At5g26930	0.7	9.6	13.5	C2C2(Zn)GATA	GATA-23	Controls lateral root founder cell specification	2
At4g26930	0.4	4.6	13.0	MYB	MYB97	Unknown function	5
At1g48000	1.3	13.8	11.1	MYB	MYB112	Unknown function	5
At5g51190	1.9	18.5	9.9	AP2/EREBP	–	Unknown function	5
At5g43540	0.4	3.2	8.8	C2H2	C1-1iAf-03	Unknown function	5
At3g55980	1.9	15.7	8.4	C3H	SZF1	Regulates salt stress responses	4
At1g74080	0.5	4.0	8.3	MYB	MYB122	Activator of the indole glucosinolate biosynthesis	4
At1g68880	0.6	5.1	8.1	bZIP	bZIP8	Unknown function	5
At4g35900	1.0	7.5	8.0	bZIP	bZIP14/FD-1	Required for regulation of flowering	2
At1g30135	0.8	5.9	7.6	ZIM	JAZ8	Represses jasmonate-regulated growth and defense responses	3
At4g01350	0.6	4.6	7.5	CHP-rich	–	Intracellular signal transduction, oxidation-reduction process, response to chitin	4
At1g43160	1.2	8.8	7.5	AP2/EREBP	RAP2.6	Regulation of development	2
At5g26170	0.8	6.3	7.5	WRKY	WRKY50	Repression of jasmonate-mediated signaling	3
At1g29280	0.8	5.5	7.2	WRKY	WRKY65	Unknown function	5
At1g75540	0.8	5.2	6.8	C2C2(Zn)CO	STH2	Positive regulation of photomorphogenesis	4
At2g33710	1.9	11.4	5.9	AP2/EREBP	ERF112	Unknown function	5
At3g01600	0.6	3.6	5.8	NAC	ANAC044	Unknown function	5
At5g27050	1.4	8.2	5.7	MADS	AGL101	Unknown function	5
At5g01380	0.9	5.3	5.7	Trihelix	–	Unknown function	5
At1g65130	1.2	6.4	5.5	C2H2	C2-1iB-03	Unknown function	5
At5g23260	1.0	5.4	5.4	MADS	AGL32/TT16	Regulates proanthocyanidin biosynthesis	1
At3g11580	0.9	4.6	5.4	ABI3/VP1	AP2/B3-like	Seed development	2
At3g56770	0.8	4.5	5.3	bHLH	–	Unknown function	5
At1g65110	0.6	3.2	5.1	C2H2	C2-1iB-01	Unknown function	5
At2g47190	1.2	6.0	4.9	MYB	MYB2	Inhibits cytokinin-mediated branching at late stages of development	2
At5g52260	1.0	4.7	4.8	MYB	MYB19	Unknown function	5
At5g39610	1.1	5.5	4.8	NAC	ANAC092/ORE1	Regulator of leaf senescence	2
At4g18880	1.6	7.4	4.6	HSF	HsfA4a/HSF21	Unknown function	5
At4g37610	1.1	4.8	4.3	TAZ	BTB5	Unknown function	5
At1g18960	1.3	5.4	4.3	MYB	–	Unknown function	5
At5g02470	0.8	3.2	4.0	E2F/DP	DPA	Endoreduplication control	2
At5g26880	1.0	3.8	3.9	MADS	AGL26	Unknown function	5
At1g68800	0.9	3.5	3.8	TCP	TCP12/BRC2	Prevents axillary bud development and outgrowth	2
At5g07500	1.9	7.0	3.7	C3H	C3H54	Required for heart-stage embryo formation	2
At4g01540	1.3	4.2	3.4	NAC	ANAC068	Mediates cytokinin signaling during cell division	2
At5g51780	1.6	5.4	3.3	bHLH	–	Unknown function	5
At2g42150	1.1	3.5	3.2	BD	–	Unknown function	5
At5g13220	1.1	3.4	3.2	ZIM	JAZ10/TIFY9	Jasmonate signaling repressor	3
At5g22290	1.2	3.8	3.1	NAC	ANAC089	Negative regulator of floral initiation	2
At2g13150	1.0	3.1	3.1	bZIP	bZIP31	Unknown function	5
At1g70700	1.1	3.3	3.0	ZIM	JAZ9	Jasmonate signaling repressor	3
At5g62320	1.2	3.5	3.0	MYB	MYB99	Unknown function	5
At4g39070	1.2	3.5	2.9	C2C2(Zn)CO	DBB2	Unknown function	5
At2g30250	1.6	4.4	2.8	WRKY	WRKY25	Involved in response to various abiotic stresses	4
At5g64810	1.7	4.8	2.8	WRKY	WRKY51	Repression of jasmonate-mediated signaling	3
At3g05800	1.9	5.3	2.7	bHLH	AIF1	Involved in brassinosteroid signaling	4
At3g01970	1.4	3.8	2.6	WRKY	WRKY45	Unknown function	5
At1g75490	1.7	4.4	2.7	AP2/EREBP	DREB2D	Unknown function	5
At1g68840	1.2	3.1	2.5	AP2/EREBP	RAV2/TEM2	Repressor of flowering	2
At1g79180	1.4	3.3	2.5	MYB	MYB63	Activates secondary wall biosynthesis	2
At4g09460	1.7	3.6	2.2	MYB	MYB8	Unknown function	5
At1g66600	1.4	3.0	2.1	WRKY	WRKY63	Involved in the regulation of responses to ABA and drought stress	4
At2g43500	1.5	3.1	2.1	NIN-like	–	Unknown function	5
At4g01520	1.8	3.7	2.0	NAC	ANAC067	Unknown function	5
At1g21000	1.6	3.2	2.0	PLATZ	–	Unknown function	5
At3g27810	1.7	3.4	2.0	MYB	MYB21	Petal and stamen development	2
At5g67300	1.6	3.1	1.9	MYB	MYB44	Regulates ethylene signaling	4
At2g39250	1.7	3.1	1.8	AP2/EREBP	SNZ	Represses flowering	2
At4g16780	1.7	3.1	1.8	HB	HB2/HAT4	Involved in cell expansion and cell proliferation	2
At4g24240	1.8	3.2	1.8	WRKY	WRKY7	Involved in plant defense responses	4
At4g01930	1.8	3.1	1.7	BPC/BRR	–	Unknown function	5
At5g62020	1.8	3.1	1.7	HSF	HsfB2a/HSF6	Unknown function	5
At2g43000	1.9	3.2	1.7	NAC	JUB1/ANAC042	Regulates camalexin biosynthesis and longevity	4
At4g17785	1.9	3.2	1.6	MYB	MYB39	Unknown function	5

Genes are listed according to the difference of the expression change between GO and wild-type (WT) plants (FC_GO_/FC_WT_). AGI: gene identification number given by the Arabidopsis Genome Initiative. A function was described for a gene when its involvement in a biological process/function was experimentally backed up as described in PubMed (www.ncbi.nlm.nih.gov/pubmed) or TAIR (http://arabidopsis.org/index.jsp). FG: functional group.

**Figure 2:**
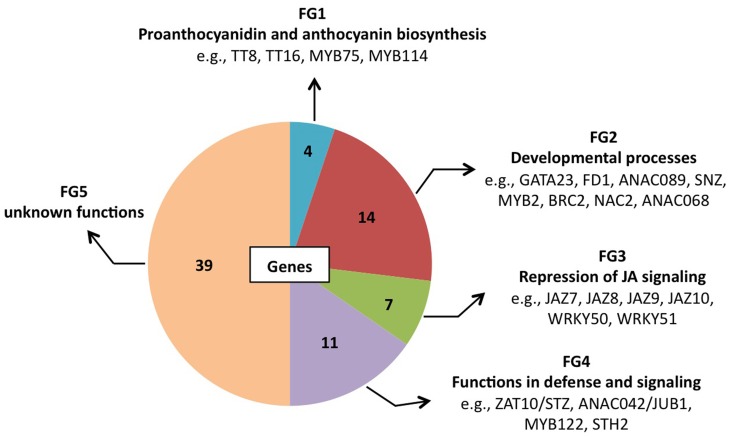
**Pie chart representation of the five functional groups (FG) of early H_2_O_2_-responsive TFs in GO plants.** FG5 includes genes for which a distinct biological function has not been reported yet.

At 0.5 h after shifting plants to ambient CO_2_ concentration, 78 of the 1449 genes were induced by more than 3-fold in GO plants, whereas in WT plants the expression changes of the same genes were less than 2-fold (Table 4). Using published data the involvement/participation of the TFs in specific biological processes (Table 4) could be assessed, which allowed the classification of the TFs into five functional groups (FG) enriched with specific gene ontology categories (Figure 2). FG1 contains TFs involved in the regulation of proanthocyanidin and anthocyanin biosynthesis (Table 4 and Figure 2). The TFs TT8 and MYB75 affecting the gene expression of dihydroflavonol 4-reductase (Debeaujon et al., 2003) are included in this FG. FG2 contains TFs affecting developmental processes like lateral root formation (GATA23), flowering (FD1, ANAC089, TEM2 and SNZ), shoot branching (MYB2 and BRC2), senescence (ANAC092/ORE1) and cell division (ANAC068 and HAT4) (Table 4 and Figure 2). The activation of these TFs in GO plants would result in altered growth and flowering (see below and Fahnenstich et al., 2008). FG3 includes TFs and TF-interacting proteins negatively regulating jasmonate (JA) signaling (*JAZ7*, *JAZ8*, *JAZ9*, *JAZ10*, *WRKY50*, and *WRKY51*; Chico et al., 2008; Staswick, 2009; Gao et al., 2011) (Table 4 and Figure 2). JAZ proteins bind directly to the key transcription factor MYC2 and thereby prevent JA-dependent gene transcription (Chini et al., 2007; Pauwels et al., 2010). At the same time *JAZ* genes are rapidly induced by JA and some are MYC2-regulated. This feedback loop regulation would provide a rapid on and off switch of the pathway involving JA. Transcriptional activation of *JAZ* genes was found to occur in response to several biotic and abiotic challenges (Yan et al., 2007). JAZ proteins would also exert their effects on post-wound inhibition of vegetative growth in *A. thaliana* (Yan et al., 2007) and as repressors of necrosis and/or programmed cell death during development in tobacco (Oh et al., 2012). In GO plants, the action of *JAZ* genes together with those of FG2 would impact growth and reproductive capacity, resulting in altered development under conditions that promote the formation of H_2_O_2_. FG4 includes TFs with diverse functions in plant defense and signaling, e.g., activators of tryptophan-derived biosynthesis of camalexin (JUB1/ANAC042) and indole glucosinolates (MYB122), as well as regulators of photomorphogenesis (STH2) (Table 4 and Figure 2). The early activation of camalexin and indole glucosinolate biosynthesis was also observed in the analysis performed with the ROS-responsive gene platform (Table 1). Finally, FG5 includes TFs with currently unknown functions (Table 4 and Figure 2).

The analysis of the transcript profiles at 0.5 h after induction of H_2_O_2_ production in GO plants (Table A2 in Appendix) also revealed a group of 13 genes that are down-regulated in GO relative to WT plants (Table 5). The function of five of these genes is currently unknown, but interestingly, the remaining eight genes positively control developmental processes. The down-regulation of expression of these TFs in GO plants together with the up-regulation of expression of TFs negatively affecting development (see FG2, Table 3) would act in concert to arrest growth and especially to delay the transition from the vegetative to the reproductive phase. Consistently, our previous results showed that GO plants growing under photorespiratory conditions are smaller than WT plants, presenting a reduced rosette diameter and a delay in flowering time (Fahnenstich et al., 2008).

**Table 5:** **Transcription factors the expression of which was reduced by more than 3-fold in GO plants 0.5 h after shifting plants grown at high CO_2_ concentration to ambient CO_2_ concentration**.

**AGI**	**0.5 h after transfer to ambient CO**_**2**_			**Gene family**	**Annotation**	**Function**
	**FC**_**WT**_	**FC**_**GO**_	**FC**_**WT**_**/FC**_**GO**_			
At3g02310	47.1	0.12	380.8	MADS	SEP2/AGL4	Flower and ovule development
At3g13850	2.0	0.02	129.5	AS2 (LOB) I	ASL30/LBD22	Unknown function
At4g00260	21.6	0.23	92.1	B3	MEE45	Maternal effect embryo arrest 45
At4g27330	2.4	0.03	78.6	NZZ	NZZ/SPL	Controls stamen identity
At1g54760	11.6	0.31	37.6	MADS	AGL85	Unknown function
At3g60460	4.6	0.26	17.9	MYB	DUO1	Plays essential role in sperm cell specification
At2g45650	3.4	0.20	17.3	MADS	AGL6/RSB1	Involved in axillary bud formation; control of flowering time and lateral organ development
At5g26950	2.0	0.17	12.2	MADS	AGL93	Unknown function
At3g15170	1.9	0.16	11.9	NAC	ANAC054/CUC1	Shoot apical meristem formation and auxin-mediated lateral root formation; formation of organ boundary
At5g58280	0.8	0.15	5.3	B3	–	Unknown function
At5g15800	1.0	0.21	5.0	MADS	SEP1/AGL2	Involved in flower and ovule development
At3g56660	1.3	0.26	5.0	bZIP	bZIP49	Unknown function
At5g23000	0.6	0.18	3.3	MYB	MYB37/RAX1	Regulates axillary meristem formation; earliest spatial marker for future axillary meristems

Genes are listed according to the difference of the expression change between wild-type (WT) and GO plants (FC_WT_/FC_GO_). A function was described for a gene when its involvement in a biological process/function was experimentally backed up as described in PubMed (www.ncbi.nlm.nih.gov/pubmed) or TAIR (http://arabidopsis.org/index.jsp).

## Concluding remarks

The metabolic induction of H_2_O_2_ formation in chloroplasts of GO plants under photorespiratory conditions triggered a faster and more prominent transcriptional response of ROS-responsive genes in these plants than in wild type. The changes of the transcriptional activities observed in GO plants early after induction of H_2_O_2_ production in chloroplasts suggest the establishment of responses that resemble those occurring at early times after wounding or herbivore attack, where H_2_O_2_ is also produced (Orozco-Cardenas and Ryan, 1999). These responses include (1) the retardation of development, which in part would be linked to JA signaling, and (2) the production of the phytoanticipins indole glucosinolates and camalexin. As in the case of herbivore attack, the retardation of development such as reductions in growth and reproduction observed in GO plants could be regarded as a strategy to allow more resource allocation to support defense and tolerance responses (Zavala and Baldwin, 2006). The data also suggest that signals for the early induction of indole glucosinolate and camalexin biosynthesis genes in GO plants through H_2_O_2_ may originate in chloroplasts as these genes showed no modified expression in catalase loss-of-function mutants (Inzé et al., 2012).

### Conflict of interest statement

The authors declare that the research was conducted in the absence of any commercial or financial relationships that could be construed as a potential conflict of interest.

## Appendix

**Table A1:** **Expression profile of 131 ROS-responsive genes in wild-type (WT) and GO plants after transferring seedlings to ambient CO_2_ concentration**.

**AGI**	**log2 FCh**							
	**0.5h WT**	**0.5h GO**	**4h WT**	**4h GO**	**6h WT**	**6h GO**	**12h WT**	**12h GO**
At1g02450	1.64	0.53	2.04	1.21	4.35	2.28	4.78	2.50
At1g05340	0.31	1.77	1.81	2.40	2.10	2.98	6.07	4.71
At1g05575	0.61	5.51	−1.36	1.90	−0.65	2.11	3.67	2.46
At1g10040	0.21	0.85	1.27	1.05	1.06	3.38	4.88	3.04
At1g13340	0.07	1.07	1.64	1.37	1.78	2.77	3.92	3.92
At1g14040	0.20	0.05	0.37	−0.24	0.42	0.28	1.54	0.51
At1g14200	−0.37	−0.58	−0.20	−1.26	0.38	0.52	1.92	1.20
At1g15520	0.22	−0.24	4.12	3.10	3.64	6.81	8.06	5.72
At1g16030	0.34	−0.29	0.11	0.71	0.18	0.13	0.95	0.22
At1g17170	−0.06	0.39	2.44	2.31	2.52	5.34	8.73	7.58
At1g17180	−0.65	−0.18	2.77	2.79	2.91	6.70	8.30	6.36
At1g18570	0.26	2.76	−1.48	−0.37	−0.53	1.33	3.99	2.48
At1g19020	0.87	3.21	0.34	1.88	0.93	4.79	6.83	5.94
At1g21100	0.72	2.78	−1.31	0.21	−0.56	0.61	1.18	1.88
At1g21120	−0.06	3.31	0.51	1.19	−0.47	3.99	4.94	3.88
At1g22400	0.08	−0.51	−0.53	−0.80	0.23	0.31	2.96	2.64
At1g26380	0.20	1.78	0.44	1.10	1.04	5.28	6.15	5.05
At1g26420	0.62	0.69	0.20	1.21	1.26	4.14	5.88	5.17
At1g27730	0.17	4.91	2.63	3.85	2.58	4.81	4.55	4.42
At1g28190	0.13	1.95	0.88	1.70	1.28	2.49	4.54	3.45
At1g35210	0.12	3.48	1.42	2.99	1.56	3.85	4.89	3.67
At1g35230	0.86	1.16	3.10	3.56	4.79	5.14	5.71	4.79
At1g53540	1.07	−0.58	3.69	2.64	1.84	0.61	3.25	2.09
At1g54050	−0.32	−0.27	1.45	0.96	0.99	0.38	−0.94	−0.35
At1g57630	0.50	1.78	3.52	3.63	4.34	5.17	7.31	5.93
At1g59590	0.34	2.37	−0.49	1.78	0.45	2.38	4.38	3.47
At1g61340	0.58	4.60	3.49	4.09	2.73	3.39	3.14	2.37
At1g61820	1.12	−0.18	0.36	−0.31	0.44	1.35	0.67	−0.26
At1g62300	0.81	1.45	1.78	2.26	2.80	3.71	4.75	4.20
At1g63720	0.63	2.31	1.45	1.89	1.65	2.61	4.47	3.77
At1g66060	0.40	4.13	0.65	1.31	1.45	2.35	3.48	3.19
At1g69890	0.40	3.99	−0.98	0.72	0.30	1.17	2.46	1.52
At1g72520	0.76	3.97	2.97	2.83	2.25	3.28	2.90	2.46
At1g73010	−1.19	−0.41	3.01	0.07	2.30	0.83	0.28	−0.72
At1g74310	−0.20	0.23	0.36	−1.58	−0.38	−0.02	0.93	0.89
At1g74360	−0.03	1.13	1.65	1.64	2.06	3.83	4.91	4.33
At1g74590	0.04	−0.20	0.60	0.27	0.58	0.26	2.51	1.39
At1g76070	0.21	1.38	0.63	1.49	0.71	1.72	2.85	2.14
At1g76600	0.44	2.54	0.51	2.42	0.81	3.45	5.35	4.45
At1g77450	−0.38	2.87	3.24	2.02	2.49	2.79	3.49	2.98
At1g80820	0.21	0.59	2.97	2.05	1.34	2.97	2.65	2.13
At1g80840	0.10	5.25	1.19	3.41	2.45	5.82	7.28	6.96
At2g15480	0.00	1.47	0.06	0.79	0.25	2.84	5.35	4.25
At2g18210	0.05	3.07	0.43	0.96	0.17	1.55	0.61	0.22
At2g18690	0.59	2.15	2.04	2.34	2.73	4.62	6.05	5.59
At2g20560	−0.17	0.21	0.92	−0.31	−0.15	0.05	2.15	1.16
At2g22470	0.09	−0.19	2.37	1.46	2.77	1.30	1.04	1.13
At2g22880	0.79	5.67	1.97	3.69	2.64	4.81	4.38	4.64
At2g24540	0.42	1.13	8.22	7.56	4.97	5.63	0.77	2.64
At2g25735	0.86	3.43	−2.23	0.62	−0.84	1.68	3.57	3.00
At2g26150	−0.59	−0.02	1.64	−0.56	0.65	−0.58	2.39	1.80
At2g26530	0.32	3.73	0.82	3.15	1.07	2.42	2.44	2.12
At2g29490	−0.54	0.94	2.15	1.53	1.22	3.38	4.92	4.77
At2g29500	−0.04	0.17	1.43	1.02	0.63	1.94	0.96	1.79
At2g31945	0.40	1.82	1.06	1.22	1.17	2.34	1.79	2.25
At2g32030	0.42	4.03	0.48	2.74	0.93	4.61	5.56	4.93
At2g32120	0.46	0.37	−0.52	−0.09	−0.06	−0.86	−0.30	0.07
At2g33710	−0.04	2.90	−0.19	1.56	−0.25	3.66	3.87	3.13
At2g37430	−0.23	5.74	1.35	4.97	0.92	7.58	5.62	5.59
At2g38250	0.25	0.77	1.49	2.30	2.15	3.77	5.55	4.43
At2g38340	−0.07	−0.61	1.12	1.13	1.44	2.60	6.22	4.70
At2g38470	0.50	4.98	0.67	2.14	0.82	2.92	4.44	3.37
At2g40000	−0.14	3.35	−0.11	0.23	0.71	1.09	1.44	1.20
At2g41380	0.19	0.27	2.95	2.64	3.26	4.41	5.92	4.88
At2g41640	0.12	2.02	0.91	1.01	1.17	2.59	2.84	2.49
At2g43820	0.50	−0.11	−1.38	−0.24	0.09	0.68	3.02	3.05
At2g47000	0.15	0.22	0.59	2.57	0.55	4.43	7.60	6.33
At3g02800	0.14	2.31	0.32	0.24	0.24	2.02	2.39	2.03
At3g02840	0.16	6.31	2.03	3.30	1.52	6.03	5.78	4.98
At3g09270	0.63	0.34	1.67	1.09	0.99	0.59	3.57	2.16
At3g09350	−0.39	−0.12	1.10	−0.76	0.47	0.18	3.05	1.71
At3g09410	0.28	−0.27	0.22	0.81	0.22	1.66	1.46	−0.32
At3g10320	−0.36	0.84	2.31	1.49	3.05	2.85	6.78	3.90
At3g10930	0.72	4.25	1.97	3.89	3.08	5.28	7.56	5.90
At3g11840	1.19	1.22	1.67	0.87	0.88	1.81	3.81	2.49
At3g12580	1.37	−1.54	2.02	−0.52	−0.04	0.17	3.40	3.04
At3g13790	0.33	0.40	1.26	1.25	1.30	2.33	3.74	4.00
At3g15950	0.61	0.49	−0.10	0.56	−0.48	−0.36	0.16	−1.29
At3g16530	0.75	1.15	−0.28	0.06	−1.28	0.54	4.65	1.65
At3g17690	0.46	−0.81	3.25	1.73	2.59	2.74	6.04	3.25
At3g23230	1.17	3.23	0.32	0.93	1.19	4.09	5.32	4.06
At3g24500	−0.21	−0.49	0.36	−1.09	−0.42	−0.04	1.97	0.95
At3g25250	0.60	4.11	3.64	5.31	3.47	7.26	8.67	7.12
At3g26830	0.15	1.47	2.83	1.84	2.11	6.03	9.59	7.36
At3g28210	0.46	0.60	1.23	2.06	1.51	1.83	3.96	3.45
At3g46230	0.47	−0.41	2.47	2.16	1.53	0.83	1.95	1.24
At3g48650	0.96	3.89	1.28	2.32	2.62	2.89	4.03	4.23
At3g49580	−0.33	0.23	0.36	1.50	1.69	2.34	−0.07	0.17
At3g53230	0.98	−0.30	1.79	0.98	2.59	1.30	3.39	2.56
At3g55980	0.51	3.84	−0.17	0.94	0.26	1.99	3.60	2.75
At4g11280	0.62	1.59	0.86	1.13	0.45	2.56	4.27	3.03
At4g12400	0.07	−0.19	0.03	−0.76	−0.78	−0.16	1.54	0.28
At4g15975	0.79	0.36	−0.56	1.46	0.64	1.89	2.25	1.54
At4g17490	0.54	4.42	−0.53	2.52	0.41	2.78	3.29	2.20
At4g18880	0.71	2.71	0.86	2.34	0.92	2.21	3.85	2.85
At4g21390	0.88	3.70	2.50	3.44	2.71	4.86	4.60	4.41
At4g21990	−0.31	0.65	1.41	0.59	0.41	1.39	1.54	0.92
At4g22530	0.38	−0.24	0.47	1.22	1.28	2.57	5.23	3.65
At4g24160	0.26	0.81	0.44	0.42	0.93	1.16	2.30	1.45
At4g24380	0.35	1.30	1.66	2.38	1.72	1.98	1.16	1.32
At4g24570	0.47	4.23	0.83	1.58	1.84	1.74	3.07	2.49
At4g37370	0.49	2.81	1.93	3.87	1.49	6.08	7.75	6.09
At4g39670	0.42	3.84	4.90	4.92	4.99	7.53	7.85	7.52
At5g04340	−0.25	4.78	3.28	3.13	2.87	3.10	5.35	4.22
At5g05730	0.52	0.78	1.40	2.23	0.49	1.27	2.95	1.99
At5g12020	0.23	−0.51	5.32	2.84	4.18	2.59	3.09	4.15
At5g12030	−0.18	0.07	3.39	1.03	1.22	1.97	2.90	3.00
At5g14700	0.19	1.14	2.48	2.11	1.59	1.74	1.49	0.80
At5g14730	0.43	0.40	−2.54	0.66	−1.85	1.42	3.48	2.00
At5g20230	0.60	1.47	2.40	3.33	3.30	4.82	6.06	5.41
At5g24110	−0.06	3.73	3.44	2.57	1.70	6.51	5.79	5.52
At5g25450	0.56	0.10	0.28	0.71	−0.36	0.63	3.48	1.90
At5g26220	0.28	−0.92	3.63	3.72	3.07	3.70	1.03	2.45
At5g27420	0.31	5.03	1.59	2.62	1.58	3.90	5.10	4.47
At5g28630	0.49	2.02	−2.90	−1.34	−1.83	−1.21	0.61	−0.72
At5g35320	−0.22	−0.13	0.67	−0.22	0.35	0.34	1.39	0.90
At5g46080	0.08	0.91	0.12	0.83	0.24	1.88	2.22	1.80
At5g47230	0.33	4.12	−0.05	2.21	0.55	2.13	3.61	2.44
At5g48570	0.33	−0.17	0.75	−0.27	−0.33	0.15	1.24	0.09
At5g48850	0.08	−0.39	1.07	1.52	1.74	2.90	−0.01	−0.41
At5g51060	0.40	−0.06	3.50	1.86	3.84	5.46	3.43	3.85
At5g51190	0.48	2.77	0.08	2.40	0.88	2.31	3.88	2.30
At5g51440	0.34	−0.29	0.65	1.16	−0.44	3.85	7.36	6.33
At5g52640	0.06	−0.18	0.96	−0.98	0.22	1.21	2.80	2.26
At5g54490	0.56	2.96	0.57	2.85	0.59	2.05	4.01	3.20
At5g57220	1.48	3.18	−1.68	0.44	−1.37	2.76	4.81	3.61
At5g59820	0.03	3.84	2.65	4.19	2.19	5.95	5.87	4.99
At5g63790	−0.37	2.42	1.55	1.69	1.63	3.47	3.56	3.23
At5g64310	0.29	1.92	1.58	1.13	1.61	0.13	0.53	0.54
At5g64510	−0.16	0.22	1.38	0.09	0.88	0.80	1.18	1.12
At5g64905	0.44	2.58	1.89	2.56	1.04	5.04	6.27	4.05

Numbers indicate log2 fold-change (FCh) expression ratio of genes after transferring plants to ambient CO_2_ concentration compared to high CO_2_ concentration. Values are means of two biological replicates.

**Table A2:** **Numbers indicate log2 fold-change (FCh) expression ratio of genes after transferring plants to ambient CO_2_ concentration compared to high CO_2_ concentration**.

**AGI**	**Gene family**	**log2 FCh**	
		**0.5h WT**	**0.5h GO**
At1g01030	ABI3/VP1	0.74	0.97
At3g16280	AP2/EREBP	0.34	0.49
At3g16770	AP2/EREBP	0.28	0.61
At2g30470	ABI3/VP1	−0.01	−0.02
At3g20310	AP2/EREBP	0.82	0.98
At2g36080	ABI3/VP1	0.53	1.08
At2g46870	ABI3/VP1	−0.03	−0.16
At3g11580	ABI3/VP1	−0.23	2.19
At3g23230	AP2/EREBP	1.28	4.67
At3g25730	AP2/EREBP	0.05	0.61
At3g61970	ABI3/VP1	−0.11	0.10
At3g25890	AP2/EREBP	0.18	0.54
At4g01500	ABI3/VP1	0.92	0.50
At3g50260	AP2/EREBP	4.28	4.54
At4g21550	ABI3/VP1	−1.81	−3.72
At3g54320	AP2/EREBP	0.63	0.32
At5g60450	ARF	0.02	−0.28
At3g18990	B3	−0.02	0.53
At5g62000	ARF	0.38	0.47
At3g46770	B3	−1.55	0.31
At1g04880	ARID	0.01	−0.59
At3g53310	B3	−0.46	0.31
At1g20910	ARID	0.22	0.32
At4g00260	B3	4.43	−2.09
At4g01580	B3	−0.21	0.06
At1g76110	ARID	0.00	0.43
At1g76510	ARID	0.03	0.81
At4g31620	B3	1.67	−0.99
At2g46040	ARID	0.14	0.29
At3g13350	ARID	−0.31	0.31
At3g43240	ARID	−0.01	0.03
At4g31650	B3	−0.28	−0.17
At4g32010	ABI3/VP1	0.36	0.21
At5g06250	ABI3/VP1	−0.53	−0.16
At3g57600	AP2/EREBP	0.24	0.25
At1g14510	Alfin	0.10	0.17
At3g60490	AP2/EREBP	0.06	1.23
At2g02470	Alfin	0.29	0.45
At3g61630	AP2/EREBP	0.68	0.94
At3g11200	Alfin	−0.15	0.33
At4g06746	AP2/EREBP	1.76	3.06
At3g42790	Alfin	−0.01	0.42
At4g11140	AP2/EREBP	−0.16	−0.03
At5g05610	Alfin	0.10	0.51
At4g13040	AP2/EREBP	0.29	0.88
At5g20510	Alfin	−0.54	0.10
At5g26210	Alfin	−0.11	0.58
At4g16750	AP2/EREBP	−0.56	1.55
At1g01250	AP2/EREBP	−0.28	0.58
At4g17490	AP2/EREBP	4.25	5.93
At1g03800	AP2/EREBP	1.34	0.43
At4g17500	AP2/EREBP	1.21	2.79
At1g07900	AS2 (LOB) I	1.44	2.66
At1g16530	AS2 (LOB) I	1.40	0.91
At4g33280	B3	0.70	0.00
At1g31320	AS2 (LOB) I	0.77	0.35
At4g34400	B3	2.13	−0.37
At1g65620	AS2 (LOB) I	0.21	0.09
At5g18000	B3	−1.97	−0.43
At5g18090	B3	−0.20	−0.09
At5g32460	B3	0.41	6.95
At2g19820	AS2 (LOB) I	0.64	1.07
At5g58280	B3	−0.34	−2.35
At2g28500	AS2 (LOB) I	2.53	4.01
At5g60130	B3	−1.04	0.36
At5g60140	B3	0.34	0.37
At2g30340	AS2 (LOB) I	0.19	0.81
At1g06160	AP2/EREBP	−0.34	1.14
At4g23750	AP2/EREBP	0.75	0.56
At4g25470	AP2/EREBP	6.18	6.85
At1g12630	AP2/EREBP	−0.01	0.40
At4g25480	AP2/EREBP	6.17	5.68
At1g12890	AP2/EREBP	1.89	2.51
At4g27950	AP2/EREBP	0.73	−0.08
At1g13260	AP2/EREBP	0.63	1.06
At4g28140	AP2/EREBP	5.79	8.83
At4g31060	AP2/EREBP	−0.16	−0.16
At1g16060	AP2/EREBP	0.22	0.42
At4g32800	AP2/EREBP	−0.09	0.94
At1g19210	AP2/EREBP	3.96	4.18
At4g34410	AP2/EREBP	6.02	8.13
At1g21910	AP2/EREBP	−0.50	0.27
At4g36900	AP2/EREBP	0.31	1.15
At1g22190	AP2/EREBP	5.46	2.69
At4g36920	AP2/EREBP	−0.12	0.06
At1g22810	AP2/EREBP	6.10	7.03
At4g37750	AP2/EREBP	0.16	0.01
At1g01260	bHLH	0.40	0.98
At2g40470	AS2 (LOB) I	−0.03	1.14
At1g02340	bHLH	−0.38	0.48
At1g03040	bHLH	0.05	0.42
At1g05805	bHLH	0.26	0.69
At1g06150	bHLH	0.09	−0.14
At2g45420	AS2 (LOB) I	−0.51	1.14
At1g06170	bHLH	−0.71	0.30
At1g09250	bHLH	−0.08	0.96
At3g11090	AS2 (LOB) I	−0.12	0.14
At1g09530	bHLH	0.01	0.32
At3g13850	AS2 (LOB) I	1.00	−6.02
At1g10120	bHLH	−0.24	−0.09
At3g26620	AS2 (LOB) I	0.44	1.02
At1g10610	bHLH	−0.31	−0.23
At3g27650	AS2 (LOB) I	−0.85	0.20
At1g12860	bHLH	−0.38	0.00
At1g22985	AP2/EREBP	−0.01	0.73
At4g39780	AP2/EREBP	1.31	1.31
At5g05410	AP2/EREBP	2.04	2.95
At1g25560	AP2/EREBP	−0.64	0.28
At5g07580	AP2/EREBP	−1.35	−0.87
At5g10510	AP2/EREBP	0.85	−0.42
At1g28360	AP2/EREBP	1.36	0.70
At5g11190	AP2/EREBP	−0.20	0.55
At1g28370	AP2/EREBP	4.77	6.09
At5g11590	AP2/EREBP	0.72	1.28
At1g33760	AP2/EREBP	2.49	3.60
At5g13330	AP2/EREBP	0.32	0.69
At1g36060	AP2/EREBP	1.26	0.20
At5g13910	AP2/EREBP	−0.26	−0.89
At1g43160	AP2/EREBP	0.23	3.13
At1g44830	AP2/EREBP	−2.15	−0.73
At1g46768	AP2/EREBP	1.17	1.07
At3g27940	AS2 (LOB) I	−0.16	0.39
At1g18400	bHLH	−0.34	0.85
At1g22490	bHLH	0.09	0.48
At3g50510	AS2 (LOB) I	−0.42	−0.90
At4g00210	AS2 (LOB) I	−0.04	−0.62
At1g26260	bHLH	0.27	0.61
At4g00220	AS2 (LOB) I	0.19	0.64
At1g27660	bHLH	−0.17	0.58
At4g22700	AS2 (LOB) I	2.94	6.51
At1g29950	bHLH	−0.04	0.21
At5g63090	AS2 (LOB) I	−0.61	1.16
At1g31050	bHLH	−0.13	0.66
At1g32640	bHLH	1.91	2.86
At1g35460	bHLH	0.12	0.70
At5g19790	AP2/EREBP	−2.72	4.75
At1g50640	AP2/EREBP	1.59	1.23
At5g25190	AP2/EREBP	0.01	0.99
At5g25390	AP2/EREBP	−1.43	1.04
At5g25810	AP2/EREBP	−0.56	0.18
At1g53170	AP2/EREBP	2.63	3.00
At1g53910	AP2/EREBP	0.13	0.61
At5g44210	AP2/EREBP	1.55	1.59
At5g47220	AP2/EREBP	1.15	2.75
At1g63040	AP2/EREBP	1.40	1.52
At5g47230	AP2/EREBP	4.34	4.94
At1g64380	AP2/EREBP	4.20	4.16
At1g68550	AP2/EREBP	0.10	−0.13
At5g51190	AP2/EREBP	0.91	4.21
At1g68840	AP2/EREBP	0.32	1.62
At1g43770	bHLH	−0.44	0.19
At3g02550	AS2 (LOB) II	0.40	−0.76
At3g49940	AS2 (LOB) II	1.10	1.64
At1g51070	bHLH	0.12	0.63
At4g37540	AS2 (LOB) II	0.47	1.39
At1g51140	bHLH	−0.08	0.61
At5g67420	AS2 (LOB) II	1.15	1.20
At1g59640	bHLH	0.02	−0.10
At1g04100	Aux/IAA	−0.74	0.52
At1g04240	Aux/IAA	−0.77	0.28
At1g62975	bHLH	0.22	0.71
At1g04250	Aux/IAA	−0.33	0.99
At1g63650	bHLH	−0.09	0.44
At1g04550	Aux/IAA	0.18	0.50
At1g15050	Aux/IAA	0.46	0.92
At1g68240	bHLH	0.35	0.59
At1g15580	Aux/IAA	0.12	1.26
At1g68810	bHLH	−0.02	0.74
At1g51950	Aux/IAA	−0.20	0.45
At1g68920	bHLH	−0.18	0.15
At1g71130	AP2/EREBP	−0.06	0.73
At5g52020	AP2/EREBP	1.59	2.60
At5g53290	AP2/EREBP	0.94	−0.17
At1g71520	AP2/EREBP	3.60	5.02
At5g57390	AP2/EREBP	0.39	0.17
At1g72360	AP2/EREBP	−0.55	0.43
At5g60120	AP2/EREBP	0.40	0.42
At5g61590	AP2/EREBP	−2.96	−1.76
At1g74930	AP2/EREBP	4.49	4.80
At5g61600	AP2/EREBP	2.71	3.70
At1g75490	AP2/EREBP	0.73	2.15
At5g61890	AP2/EREBP	1.25	1.22
At1g77200	AP2/EREBP	−0.98	0.45
At5g64750	AP2/EREBP	2.48	2.27
At1g77640	AP2/EREBP	0.09	−0.48
At5g65130	AP2/EREBP	0.56	0.64
At1g78080	AP2/EREBP	2.43	2.50
At5g65510	AP2/EREBP	−0.63	−1.49
At1g79700	AP2/EREBP	−0.22	0.66
At1g52830	Aux/IAA	−0.12	0.08
At1g69010	bHLH	0.15	0.79
At1g80390	Aux/IAA	1.08	1.72
At2g01200	Aux/IAA	0.54	1.25
At1g72210	bHLH	1.02	−0.58
At2g22670	Aux/IAA	0.01	0.19
At1g73830	bHLH	−1.22	−0.72
At2g33310	Aux/IAA	0.04	0.16
At2g46990	Aux/IAA	0.07	1.22
At3g04730	Aux/IAA	−0.10	0.48
At3g15540	Aux/IAA	−0.25	0.47
At2g18300	bHLH	−0.68	0.05
At3g16500	Aux/IAA	0.10	0.71
At2g20095	bHLH	−0.12	−0.12
At3g17600	Aux/IAA	−0.27	1.57
At2g20180	bHLH	−0.53	−0.23
At3g23030	Aux/IAA	0.77	1.53
At3g23050	Aux/IAA	−0.39	0.82
At5g67180	AP2/EREBP	−0.23	−0.32
At2g20880	AP2/EREBP	5.11	7.22
At5g67190	AP2/EREBP	0.53	1.47
At2g22200	AP2/EREBP	2.28	3.07
At1g19220	ARF	0.14	−0.07
At2g23340	AP2/EREBP	0.86	0.80
At1g19850	ARF	0.49	−0.37
At2g25820	AP2/EREBP	0.00	1.42
At1g30330	ARF	−0.01	0.08
At2g28550	AP2/EREBP	0.34	0.56
At2g31230	AP2/EREBP	0.80	1.51
At2g33710	AP2/EREBP	0.95	3.51
At2g35700	AP2/EREBP	0.17	0.93
At2g38340	AP2/EREBP	0.45	−0.20
At2g39250	AP2/EREBP	0.76	1.64
At3g62100	Aux/IAA	0.32	0.57
At2g22770	bHLH	−0.12	0.93
At4g14550	Aux/IAA	−0.28	0.76
At2g24260	bHLH	0.79	0.38
At4g14560	Aux/IAA	0.83	1.05
At2g27230	bHLH	−0.04	0.21
At4g28640	Aux/IAA	−0.11	0.17
At2g28160	bHLH	−0.42	0.10
At4g29080	Aux/IAA	−0.18	0.62
At4g32280	Aux/IAA	−1.14	0.38
At5g25890	Aux/IAA	0.21	1.03
At2g31220	bHLH	−0.84	−0.45
At5g43700	Aux/IAA	−0.45	0.70
At2g31280	bHLH	0.10	0.00
At5g65670	Aux/IAA	−0.05	0.63
At2g40200	bHLH	−0.23	−0.03
At1g16640	B3	0.06	0.46
At2g41130	bHLH	0.08	1.11
At2g41240	bHLH	−0.57	−0.87
At1g59750	ARF	0.07	0.03
At2g41710	AP2/EREBP	0.18	0.38
At2g28350	ARF	−0.62	−0.35
At2g44840	AP2/EREBP	3.55	6.34
At2g33860	ARF	0.69	−1.21
At2g44940	AP2/EREBP	−1.71	0.13
At2g46530	ARF	0.16	0.35
At2g46310	AP2/EREBP	0.82	0.82
At3g61830	ARF	0.10	0.06
At2g47520	AP2/EREBP	0.66	0.34
At4g23980	ARF	0.23	0.39
At3g11020	AP2/EREBP	2.08	1.74
At4g30080	ARF	−0.25	0.49
At3g14230	AP2/EREBP	−0.02	0.72
At3g15210	AP2/EREBP	3.50	4.06
At5g37020	ARF	0.16	0.28
At1g49480	B3	−0.09	0.45
At2g42280	bHLH	0.28	0.96
At2g42300	bHLH	−0.56	−0.31
At2g24650	B3	−0.39	−0.14
At2g43010	bHLH	−0.31	0.16
At2g24680	B3	−0.08	−0.28
At2g43140	bHLH	0.57	−0.38
At2g24690	B3	−0.13	0.18
At2g46510	bHLH	0.71	1.44
At2g24700	B3	0.73	−1.17
At2g46810	bHLH	−0.30	0.56
At2g35310	B3	−0.37	−1.10
At2g46970	bHLH	−0.05	0.23
At3g06160	B3	−0.09	−0.37
At2g47270	bHLH	2.01	2.21
At3g06220	B3	0.29	−1.37
At3g05800	bHLH	0.95	2.40
At3g06120	bHLH	−0.05	0.15
At3g18960	B3	−7.62	−0.50
At3g06590	bHLH	−0.13	0.39
At3g07340	bHLH	−0.64	−0.30
At1g03970	bZIP	0.13	0.69
At3g17100	bHLH	−0.45	0.12
At1g06070	bZIP	0.42	0.19
At3g19500	bHLH	−0.26	1.20
At1g06850	bZIP	−0.43	−0.02
At3g19860	bHLH	0.41	0.25
At3g20640	bHLH	0.33	0.92
At1g13600	bZIP	−1.07	0.97
At3g21330	bHLH	0.35	0.61
At1g19490	bZIP	0.84	0.90
At3g22100	bHLH	1.18	3.16
At1g22070	bZIP	−0.40	0.67
At3g23210	bHLH	0.99	0.86
At1g32150	bZIP	0.47	0.72
At3g23690	bHLH	−0.16	0.26
At3g24140	bHLH	0.40	0.37
At3g25710	bHLH	0.75	0.89
At1g43700	bZIP	−0.04	0.56
At2g24790	C2C2(Zn) CO-like	0.18	0.97
At1g65110	C2H2	−0.67	1.69
At2g31380	C2C2(Zn) CO-like	0.59	0.81
At2g33500	C2C2(Zn) CO-like	0.11	0.65
At1g01930	C2H2	0.09	0.61
At2g47890	C2C2(Zn) CO-like	1.09	0.50
At1g02030	C2H2	0.55	0.82
At1g02040	C2H2	−0.50	0.39
At3g07650	C2C2(Zn) CO-like	0.87	1.08
At1g03840	C2H2	−0.66	−0.15
At3g21150	C2C2(Zn) CO-like	2.51	2.93
At1g04445	C2H2	2.53	0.68
At3g21880	C2C2(Zn) CO-like	2.16	2.79
At1g04990	C2H2	0.57	0.90
At3g21890	C2C2(Zn) CO-like	2.59	3.55
At1g08290	C2H2	0.59	0.38
At1g11490	C2H2	−0.51	0.33
At3g26744	bHLH	0.13	0.20
At1g45249	bZIP	0.80	1.01
At3g47640	bHLH	0.72	0.61
At1g49720	bZIP	0.49	0.83
At1g58110	bZIP	0.45	0.86
At3g56220	bHLH	−1.25	0.07
At1g68640	bZIP	−1.16	0.31
At3g56770	bHLH	−0.24	2.17
At1g68880	bZIP	−0.67	2.34
At3g56970	bHLH	0.11	0.05
At1g75390	bZIP	−0.43	−0.27
At3g56980	bHLH	0.00	−1.18
At1g77920	bZIP	−0.42	1.26
At3g57800	bHLH	−0.13	0.19
At2g04038	bZIP	−0.54	−0.49
At3g59060	bHLH	0.26	0.45
At3g61950	bHLH	−0.02	−0.58
At3g62090	bHLH	−1.46	1.29
At2g13150	bZIP	0.01	1.62
At4g27310	C2C2(Zn) CO-like	0.45	0.48
At4g38960	C2C2(Zn) CO-like	0.48	1.05
At1g14580	C2H2	2.14	0.20
At4g39070	C2C2(Zn) CO-like	0.25	1.79
At1g24625	C2H2	−0.61	0.31
At5g15840	C2C2(Zn) CO-like	0.78	0.61
At1g24630	C2H2	−0.54	0.33
At5g15850	C2C2(Zn) CO-like	0.53	1.05
At1g25250	C2H2	0.37	0.91
At5g24930	C2C2(Zn) CO-like	0.28	0.89
At1g26590	C2H2	−1.62	0.50
At5g48250	C2C2(Zn) CO-like	−0.69	0.48
At1g26610	C2H2	0.12	1.22
At5g54470	C2C2(Zn) CO-like	1.52	1.54
At1g27730	C2H2	0.82	5.18
At5g57660	C2C2(Zn) CO-like	0.19	0.82
At1g07640	C2C2(Zn) DOF	0.08	−0.17
At1g29600	C2H2	0.08	−0.17
At1g30970	C2H2	0.68	−0.31
At1g26790	C2C2(Zn) DOF	3.01	−0.64
At1g34370	C2H2	0.69	0.88
At4g00050	bHLH	0.72	0.13
At2g16770	bZIP	−0.20	0.52
At2g17770	bZIP	0.58	−0.17
At4g00480	bHLH	−0.33	0.45
At2g18160	bZIP	−0.71	−0.12
At4g00870	bHLH	0.28	−0.84
At2g21230	bZIP	−0.06	0.48
At4g01460	bHLH	0.42	0.87
At4g02590	bHLH	0.46	0.34
At2g22850	bZIP	0.04	0.88
At4g05170	bHLH	−0.31	0.75
At2g31370	bZIP	−0.02	0.42
At4g09180	bHLH	−0.09	0.20
At4g09820	bHLH	−0.41	5.10
At2g35530	bZIP	0.91	0.48
At4g14410	bHLH	0.02	0.67
At2g36270	bZIP	1.10	−1.17
At4g16430	bHLH	0.11	0.77
At2g40620	bZIP	0.26	0.07
At4g17880	bHLH	−0.77	0.12
At2g40950	bZIP	0.28	0.42
At1g28310	C2C2(Zn) DOF	−0.03	0.26
At1g29160	C2C2(Zn) DOF	0.11	0.40
At1g43850	C2H2	0.68	0.58
At1g43860	C2H2	−0.09	0.69
At1g47655	C2C2(Zn) DOF	0.24	0.22
At1g47860	C2H2	0.41	0.36
At1g51700	C2C2(Zn) DOF	1.89	2.29
At1g64620	C2C2(Zn) DOF	0.58	0.47
At1g55110	C2H2	1.08	1.07
At1g69570	C2C2(Zn) DOF	−0.38	0.90
At1g65120	C2H2	0.37	0.81
At2g28510	C2C2(Zn) DOF	0.91	0.81
At1g65130	C2H2	0.21	2.67
At2g28810	C2C2(Zn) DOF	0.55	0.14
At1g66140	C2H2	−0.06	0.25
At2g34140	C2C2(Zn) DOF	−0.27	0.52
At1g67030	C2H2	0.79	0.65
At2g37590	C2C2(Zn) DOF	0.52	0.87
At1g68130	C2H2	0.45	0.38
At2g46590	C2C2(Zn) DOF	−0.20	0.11
At1g68360	C2H2	−0.13	0.27
At4g20970	bHLH	−0.07	0.90
At2g41070	bZIP	0.53	0.53
At2g42380	bZIP	−0.86	0.03
At2g46270	bZIP	1.20	1.64
At3g10800	bZIP	0.40	0.74
At4g25410	bHLH	−0.12	0.27
At3g12250	bZIP	0.20	0.05
At4g28790	bHLH	−0.73	0.03
At3g17609	bZIP	0.95	1.16
At3g19290	bZIP	0.69	0.77
At4g29100	bHLH	0.23	0.41
At4g29930	bHLH	0.81	1.31
At4g30180	bHLH	−0.72	1.33
At3g51960	bZIP	0.19	0.90
At4g30980	bHLH	1.72	0.14
At3g54620	bZIP	0.29	0.62
At3g21270	C2C2(Zn) DOF	−0.33	0.48
At1g68480	C2H2	1.49	−0.70
At3g45610	C2C2(Zn) DOF	0.40	0.68
At1g72050	C2H2	0.28	0.53
At3g47500	C2C2(Zn) DOF	0.49	0.62
At1g75710	C2H2	0.00	0.15
At3g50410	C2C2(Zn) DOF	−0.05	0.48
At3g52440	C2C2(Zn) DOF	1.88	5.20
At2g01940	C2H2	−0.65	0.35
At3g55370	C2C2(Zn) DOF	0.29	0.63
At2g02070	C2H2	1.78	0.18
At3g61850	C2C2(Zn) DOF	−0.38	0.07
At2g02080	C2H2	0.73	0.09
At4g00940	C2C2(Zn) DOF	−0.37	0.42
At2g24500	C2H2	0.08	0.74
At3g56660	bZIP	0.37	−1.96
At4g34530	bHLH	0.09	0.38
At3g56850	bZIP	0.60	0.69
At4g36060	bHLH	0.04	0.50
At3g58120	bZIP	−1.52	−0.95
At4g36540	bHLH	−0.04	0.55
At3g62420	bZIP	−0.33	0.61
At4g36930	bHLH	0.44	−0.33
At4g01120	bZIP	0.23	0.87
At4g02640	bZIP	−0.01	0.50
At4g34000	bZIP	1.11	1.02
At5g01310	bHLH	0.43	0.70
At4g34590	bZIP	−0.18	0.39
At4g35040	bZIP	−0.47	0.57
At5g08130	bHLH	1.02	0.38
At4g35900	bZIP	−0.08	2.91
At5g09460	bHLH	−0.34	0.36
At4g36730	bZIP	0.26	0.51
At4g37730	bZIP	0.45	0.62
At4g24060	C2C2(Zn) DOF	−0.08	0.64
At2g26940	C2H2	0.02	0.88
At4g38000	C2C2(Zn) DOF	−0.99	1.49
At2g27100	C2H2	0.54	−0.21
At5g02460	C2C2(Zn) DOF	0.21	0.68
At5g39660	C2C2(Zn) DOF	0.40	1.33
At2g28200	C2H2	0.07	0.17
At5g60200	C2C2(Zn) DOF	0.27	0.77
At2g28710	C2H2	−0.51	0.59
At5g60850	C2C2(Zn) DOF	0.19	0.86
At2g29660	C2H2	−0.46	0.39
At5g62430	C2C2(Zn) DOF	0.67	1.18
At2g32930	C2H2	1.70	0.92
At5g62940	C2C2(Zn) DOF	0.06	0.71
At2g36480	C2H2	1.21	0.13
At5g65590	C2C2(Zn) DOF	−0.42	1.18
At2g36930	C2H2	0.44	0.09
At5g66940	C2C2(Zn) DOF	−0.38	−0.49
At2g37430	C2H2	0.89	6.33
At1g08000	C2C2(Zn) GATA	0.96	0.62
At1g08010	C2C2(Zn) GATA	1.05	0.41
At2g41940	C2H2	−0.76	−0.76
At5g10570	bHLH	−0.02	0.94
At4g38900	bZIP	0.12	0.61
At5g15160	bHLH	−0.19	0.27
At5g04840	bZIP	−0.06	0.25
At5g38860	bHLH	−0.35	0.30
At5g06950	bZIP	−3.42	0.81
At5g39860	bHLH	0.12	0.52
At5g06960	bZIP	−0.07	0.40
At5g41315	bHLH	1.22	1.01
At5g10030	bZIP	0.35	0.31
At5g46690	bHLH	−0.39	−0.34
At5g11260	bZIP	0.99	1.14
At5g46760	bHLH	−0.05	0.14
At5g15830	bZIP	−0.61	0.39
At5g46830	bHLH	−1.47	0.32
At5g24800	bZIP	0.20	1.07
At5g48560	bHLH	0.03	0.29
At5g28770	bZIP	−0.12	0.14
At1g51600	C2C2(Zn) GATA	0.23	0.85
At2g18380	C2C2(Zn) GATA	0.38	0.38
At2g45120	C2H2	0.20	0.28
At2g28340	C2C2(Zn) GATA	0.97	−0.02
At2g45050	C2C2(Zn) GATA	−0.39	0.90
At3g01460	C2H2	1.21	0.34
At3g06740	C2C2(Zn) GATA	−1.20	−0.64
At3g02790	C2H2	0.01	0.28
At3g16870	C2C2(Zn) GATA	0.02	−0.47
At3g02830	C2H2	0.22	0.54
At3g05760	C2H2	0.05	0.63
At3g21175	C2C2(Zn) GATA	0.07	0.55
At3g24050	C2C2(Zn) GATA	0.60	0.45
At3g10470	C2H2	0.41	−0.30
At3g13810	C2H2	0.29	1.00
At3g50870	C2C2(Zn) GATA	0.44	−0.02
At3g14740	C2H2	0.23	0.41
At3g51080	C2C2(Zn) GATA	0.43	0.39
At3g19580	C2H2	2.44	2.95
At5g50010	bHLH	−0.53	0.99
At5g50915	bHLH	0.50	1.15
At5g51780	bHLH	0.72	2.43
At5g44080	bZIP	0.05	0.31
At5g51790	bHLH	−0.57	0.21
At5g49450	bZIP	−0.04	1.02
At5g60830	bZIP	2.50	1.55
At5g54680	bHLH	0.45	0.44
At5g65210	bZIP	−0.10	0.17
At5g56960	bHLH	2.39	3.26
At1g19350	BZR	0.89	0.72
At5g57150	bHLH	0.27	0.55
At1g75080	BZR	1.20	0.95
At1g78700	BZR	1.74	0.50
At5g61270	bHLH	0.44	0.46
At3g50750	BZR	−0.10	0.34
At5g62610	bHLH	0.28	0.85
At4g18890	BZR	0.53	0.42
At5g64340	bHLH	−0.31	−0.18
At4g36780	BZR	0.65	0.53
At3g54810	C2C2(Zn) GATA	−0.02	−0.20
At3g60530	C2C2(Zn) GATA	−0.45	0.31
At4g17570	C2C2(Zn) GATA	−0.05	0.49
At4g24470	C2C2(Zn) GATA	0.48	0.09
At4g26150	C2C2(Zn) GATA	0.65	0.37
At3g44750	C2H2	0.06	0.23
At4g32890	C2C2(Zn) GATA	0.18	−0.17
At3g45260	C2H2	−0.02	0.11
At4g34680	C2C2(Zn) GATA	0.23	0.40
At4g36240	C2C2(Zn) GATA	0.02	0.26
At3g46080	C2H2	1.17	3.27
At4g36620	C2C2(Zn) GATA	0.51	0.01
At3g46090	C2H2	2.29	3.65
At5g25830	C2C2(Zn) GATA	−0.11	−0.10
At3g47890	C2H2	−0.01	0.20
At5g26930	C2C2(Zn) GATA	−0.49	3.26
At3g49930	C2H2	0.75	0.06
At5g47140	C2C2(Zn) GATA	0.04	0.70
At3g50700	C2H2	−0.32	1.30
At5g65320	bHLH	−0.83	−0.16
At1g06040	C2C2(Zn) CO-like	0.05	0.86
At5g65640	bHLH	0.04	0.79
At1g25440	C2C2(Zn) CO-like	−0.45	0.31
At5g67060	bHLH	−0.44	0.21
At1g28050	C2C2(Zn) CO-like	−0.09	1.01
At5g67110	bHLH	0.27	−0.20
At1g49130	C2C2(Zn) CO-like	−0.17	1.01
At1g14685	BPC/BRR	0.13	0.32
At1g68120	BPC/BRR	0.25	0.02
At1g68190	C2C2(Zn) CO-like	−0.39	0.54
At2g21240	BPC/BRR	−0.01	0.58
At1g68520	C2C2(Zn) CO-like	−0.58	0.18
At2g35550	BPC/BRR	0.12	0.92
At1g73870	C2C2(Zn) CO-like	0.59	0.68
At4g01930	BPC/BRR	0.85	1.64
At1g75540	C2C2(Zn) CO-like	−0.40	2.37
At1g78600	C2C2(Zn) CO-like	0.62	0.33
At5g42520	BPC/BRR	0.34	0.40
At2g21320	C2C2(Zn) CO-like	−0.09	0.69
At5g49300	C2C2(Zn) GATA	−0.24	1.01
At3g53600	C2H2	2.47	3.37
At5g56860	C2C2(Zn) GATA	−0.18	0.00
At5g66320	C2C2(Zn) GATA	2.87	0.64
At3g57480	C2H2	0.08	0.73
At1g08465	C2C2(Zn) YABBY	−0.20	0.56
At3g57670	C2H2	0.41	0.16
At3g58070	C2H2	0.09	1.07
At3g60580	C2H2	−0.19	0.51
At2g26580	C2C2(Zn) YABBY	0.37	0.81
At3g62240	C2H2	1.63	0.15
At2g45190	C2C2(Zn) YABBY	0.77	0.16
At4g02670	C2H2	0.69	0.56
At4g00180	C2C2(Zn) YABBY	0.58	0.26
At4g12240	C2H2	−0.34	0.18
At1g13400	C2H2	−0.19	−0.76
At4g15420	C2H2	0.12	0.83
At4g16610	C2H2	0.00	0.90
At4g17810	C2H2	−0.51	0.32
At3g14020	CCAAT-HAP2	1.21	0.92
At4g25610	C2H2	0.40	−0.74
At3g20910	CCAAT-HAP2	0.32	0.82
At5g06510	CCAAT-HAP2	0.02	−0.06
At4g27240	C2H2	−0.26	−0.11
At5g12840	CCAAT-HAP2	0.72	0.84
At4g31420	C2H2	−0.12	0.80
At2g13570	CCAAT-HAP3	−0.16	0.24
At2g27470	CCAAT-HAP3	0.09	−0.03
At5g01160	C2H2	0.90	0.52
At2g37060	CCAAT-HAP3	0.13	0.74
At2g38880	CCAAT-HAP3	0.42	0.78
At5g03150	C2H2	0.34	−0.17
At1g52150	HB	1.63	−0.43
At3g46640	GARP-G2-like	−0.17	−0.95
At1g62360	HB	−2.54	−1.92
At1g62990	HB	0.07	0.92
At4g13640	GARP-G2-like	0.27	0.30
At1g69780	HB	−0.93	0.70
At1g70920	HB	0.28	1.29
At4g28610	GARP-G2-like	0.50	0.62
At1g73360	HB	−0.50	0.20
At4g37180	GARP-G2-like	0.53	1.36
At1g75410	HB	0.09	0.62
At5g05090	GARP-G2-like	−0.21	0.30
At5g06800	GARP-G2-like	0.46	0.73
At1g79840	HB	−0.06	0.26
At5g16560	GARP-G2-like	−0.68	−0.18
At2g01430	HB	0.89	−0.31
At5g03510	C2H2	−0.33	−0.01
At3g53340	CCAAT-HAP3	−1.44	2.69
At5g03740	C2H2	0.13	0.28
At4g14540	CCAAT-HAP3	−0.19	0.56
At5g04340	C2H2	3.93	5.44
At5g47640	CCAAT-HAP3	0.91	1.44
At5g04390	C2H2	−0.65	−1.44
At5g47670	CCAAT-HAP3	0.54	−0.21
At1g07980	CCAAT-HAP5	−0.06	0.39
At1g08970	CCAAT-HAP5	0.23	0.74
At5g06650	C2H2	0.41	−0.09
At1g54830	CCAAT-HAP5	0.67	0.82
At5g09740	C2H2	0.15	0.51
At1g56170	CCAAT-HAP5	0.58	0.90
At5g10970	C2H2	−1.15	−0.37
At3g12480	CCAAT-HAP5	0.65	0.46
At3g48590	CCAAT-HAP5	0.52	0.54
At5g14140	C2H2	−0.05	1.09
At5g27910	CCAAT-HAP5	−1.06	1.11
At5g38140	CCAAT-HAP5	−0.17	−0.05
At5g18240	GARP-G2-like	0.09	0.67
At5g29000	GARP-G2-like	0.10	0.92
At2g02540	HB	0.05	0.06
At5g42630	GARP-G2-like	−1.15	0.23
At2g16400	HB	0.65	0.56
At5g44190	GARP-G2-like	0.01	0.22
At5g45580	GARP-G2-like	−0.96	0.18
At2g18550	HB	−1.44	1.14
At5g59570	GARP-G2-like	0.60	0.87
At2g22430	HB	1.79	1.63
At2g22800	HB	0.69	0.49
At1g44810	GeBP	−0.70	0.32
At2g23760	HB	0.03	0.55
At1g61730	GeBP	0.28	0.49
At2g27990	HB	−1.10	0.32
At2g28610	HB	−0.64	0.68
At2g25650	GeBP	−0.03	0.63
At5g16470	C2H2	−0.01	0.53
At5g43250	CCAAT-HAP5	−0.08	0.78
At5g16540	C2H2	0.15	0.85
At5g50470	CCAAT-HAP5	1.23	0.96
At5g18550	C2H2	1.57	0.12
At5g50480	CCAAT-HAP5	1.61	0.40
At5g63470	CCAAT-HAP5	−0.08	0.94
At5g25160	C2H2	−1.33	−0.40
At5g26610	C2H2	0.09	0.31
At4g01350	CHP-rich	−0.70	2.21
At5g37890	C2H2	0.30	0.61
At5g39550	C2H2	0.42	0.01
At2g20110	CPP(Zn)	0.14	−0.03
At3g04850	CPP(Zn)	−0.01	0.08
At5g40710	C2H2	−0.13	0.95
At3g16160	CPP(Zn)	−0.23	0.67
At2g36340	GeBP	−0.40	0.23
At3g04930	GeBP	0.10	0.87
At2g34710	HB	1.04	0.32
At4g00250	GeBP	−1.42	2.65
At2g35940	HB	0.53	0.95
At4g00270	GeBP	0.08	1.08
At2g36610	HB	−3.29	1.15
At4g00270	GeBP	−0.40	0.57
At2g44910	HB	−0.16	−0.13
At4g00390	GeBP	0.62	1.34
At2g46680	HB	1.05	1.08
At3g01220	HB	0.78	1.01
At3g01470	HB	0.33	0.28
At4g25210	GeBP	0.14	0.80
At5g14280	GeBP	0.06	0.25
At3g03660	HB	0.86	0.97
At5g28040	GeBP	0.49	0.46
At3g11260	HB	0.27	−0.71
At5g28040	GeBP	0.46	0.71
At3g18010	HB	0.78	1.05
At3g22760	CPP(Zn)	−0.35	0.13
At5g43170	C2H2	0.48	1.24
At3g22780	CPP(Zn)	0.13	0.19
At5g43540	C2H2	−1.44	1.69
At4g14770	CPP(Zn)	0.27	0.00
At5g44160	C2H2	0.35	0.50
At4g29000	CPP(Zn)	0.27	0.47
At5g25790	CPP(Zn)	0.21	1.24
At5g52010	C2H2	0.11	0.44
At1g47870	E2F/DP	0.33	0.46
At2g36010	E2F/DP	0.62	0.57
At3g01330	E2F/DP	−0.37	−0.25
At5g54630	C2H2	−0.29	0.21
At3g48160	E2F/DP	−0.08	0.23
At5g56200	C2H2	−3.54	3.86
At5g02470	E2F/DP	−0.34	1.65
At5g57520	C2H2	−1.45	−0.52
At5g03415	E2F/DP	0.41	0.22
At5g59820	C2H2	1.49	4.74
At5g14960	E2F/DP	1.08	0.27
At1g05055	General Transcription	0.47	0.27
At3g19510	HB	−0.08	0.11
At4g26170	General Transcription	−0.18	0.70
At3g49530	HB	1.86	2.55
At1g01160	GIF	0.22	0.35
At4g00850	GIF	0.69	1.16
At5g28640	GIF	−0.23	0.13
At1g07520	GRAS	1.57	2.32
At3g56560	HB	1.09	2.02
At1g07530	GRAS	0.23	0.71
At3g60390	HB	0.53	1.21
At1g14920	GRAS	0.18	−0.11
At3g61150	HB	0.73	0.72
At1g21450	GRAS	0.23	1.07
At3g61890	HB	1.26	1.68
At1g50420	GRAS	0.21	0.69
At1g50600	GRAS	0.17	0.95
At4g00730	HB	0.50	0.57
At5g60470	C2H2	−0.13	1.25
At5g22220	E2F/DP	0.47	0.72
At1g73730	EIL	1.23	0.52
At5g63280	C2H2	−0.03	0.21
At2g27050	EIL	−0.16	0.35
At5g64610	C2H2	−0.18	0.52
At3g20770	EIL	0.08	0.88
At5g66730	C2H2	0.28	0.22
At5g67450	C2H2	2.15	3.36
At1g32360	C3H	0.19	0.70
At1g60700	FHA	−0.01	0.07
At1g68200	C3H	0.27	0.51
At2g19810	C3H	0.75	1.30
At3g07220	FHA	0.37	0.45
At2g25900	C3H	−0.44	0.97
At3g07260	FHA	−0.25	4.25
At2g35430	C3H	0.11	0.74
At3g54350	FHA	0.28	0.42
At1g55580	GRAS	−0.24	−1.77
At4g01520	HB	0.88	1.90
At1g63100	GRAS	−0.14	−0.04
At4g01550	HB	−0.43	1.47
At1g66350	GRAS	−0.06	0.34
At4g02560	HB	0.48	0.40
At2g01570	GRAS	0.21	0.34
At4g03250	HB	0.32	0.63
At2g04890	GRAS	−0.18	0.67
At4g04890	HB	0.38	0.19
At2g37650	GRAS	0.28	0.40
At2g45160	GRAS	0.09	0.04
At4g16780	HB	0.80	1.65
At3g03450	GRAS	−0.19	0.08
At4g17460	HB	−1.60	−0.47
At3g13840	GRAS	0.16	1.57
At3g46600	GRAS	1.92	2.71
At4g21750	HB	0.60	0.64
At3g49950	GRAS	0.50	−0.70
At2g40140	C3H	3.19	3.95
At2g41900	C3H	0.16	0.46
At1g67710	GARP-ARR-B	−0.71	0.17
At3g06410	C3H	0.98	0.96
At2g01760	GARP-ARR-B	−0.85	0.18
At3g12130	C3H	0.44	0.68
At2g25180	GARP-ARR-B	0.26	−0.07
At3g12680	C3H	0.33	0.26
At3g16857	GARP-ARR-B	0.16	−0.01
At3g19360	C3H	0.64	0.38
At3g48440	C3H	0.68	0.39
At4g16110	GARP-ARR-B	−0.01	0.37
At3g51120	C3H	−0.03	0.35
At4g31920	GARP-ARR-B	−0.21	0.10
At3g55980	C3H	0.90	3.97
At5g07210	GARP-ARR-B	1.57	0.85
At4g00305	C3H	0.46	0.93
At4g01020	C3H	0.93	0.40
At4g29190	C3H	0.72	1.36
At1g13300	GARP-G2-like	1.44	0.56
At3g50650	GRAS	0.15	−0.27
At4g29940	HB	0.07	0.46
At3g54220	GRAS	0.61	0.37
At4g32040	HB	0.01	0.84
At3g60630	GRAS	0.16	0.50
At4g32880	HB	1.66	0.24
At4g00150	GRAS	0.57	0.58
At4g32980	HB	−0.66	0.12
At4g08250	GRAS	2.11	2.28
At4g34610	HB	0.27	0.20
At4g17230	GRAS	4.77	2.92
At4g35550	HB	0.22	0.40
At4g36710	GRAS	0.22	0.75
At4g37650	GRAS	0.29	0.47
At4g36870	HB	0.11	0.05
At5g17490	GRAS	0.26	1.26
At4g37790	HB	1.19	1.58
At5g41920	GRAS	0.10	0.47
At4g40060	HB	−0.01	0.70
At5g48150	GRAS	0.42	0.67
At5g02030	HB	0.12	0.55
At5g52510	GRAS	0.08	0.98
At5g03790	HB	−0.21	0.18
At5g06420	C3H	−0.39	0.04
At1g14600	GARP-G2-like	0.32	0.97
At5g06770	C3H	0.55	0.30
At1g25550	GARP-G2-like	1.12	1.73
At5g07060	C3H	0.17	1.35
At1g32240	GARP-G2-like	0.08	0.67
At5g07500	C3H	0.91	2.80
At1g49560	GARP-G2-like	−0.29	0.60
At5g12850	C3H	1.02	0.22
At1g68670	GARP-G2-like	0.47	1.19
At5g44260	C3H	−0.37	−0.47
At1g69580	GARP-G2-like	0.03	0.48
At5g58620	C3H	1.06	1.15
At1g79430	GARP-G2-like	0.39	0.03
At4g16150	CAMTA	0.23	1.05
At2g01060	GARP-G2-like	0.56	0.68
At1g67310	CAMTA	0.52	0.74
At2g02060	GARP-G2-like	0.25	0.47
At1g67910	CAMTA	0.33	0.68
At2g03500	GARP-G2-like	0.93	−0.19
At2g22300	CAMTA	0.44	1.20
At2g20400	GARP-G2-like	0.26	0.40
At2g22900	CAMTA	−0.41	0.60
At2g20570	GARP-G2-like	0.51	0.46
At5g59450	GRAS	1.21	1.78
At5g66770	GRAS	−0.12	−0.01
At5g06710	HB	0.66	0.31
At2g06200	GRF	−1.16	−1.07
At5g11060	HB	0.00	0.76
At2g22840	GRF	0.20	−0.25
At5g11270	HB	0.13	0.33
At2g36400	GRF	0.03	0.56
At5g15150	HB	−1.34	−0.12
At2g45480	GRF	−0.05	−0.23
At3g13960	GRF	0.16	0.03
At3g52910	GRF	−0.03	0.64
At5g19520	HB	−1.33	1.17
At4g24150	GRF	0.35	0.22
At5g25220	HB	0.55	1.12
At4g37740	GRF	0.97	−0.01
At5g53660	GRF	−1.00	1.00
At5g46880	HB	1.22	0.16
At1g05230	HB	0.28	0.17
At5g60690	HB	0.21	0.33
At3g16940	CAMTA	1.12	1.06
At2g38300	GARP-G2-like	0.10	0.95
At5g09410	CAMTA	0.51	0.60
At2g40260	GARP-G2-like	0.10	0.09
At5g64220	CAMTA	0.29	0.82
At2g40970	GARP-G2-like	0.23	0.85
At5g08190	CCAAT-DR1	0.01	0.22
At5g23090	CCAAT-DR1	−0.08	0.65
At3g04030	GARP-G2-like	0.06	−0.23
At1g17590	CCAAT-HAP2	0.04	0.59
At3g04450	GARP-G2-like	−0.09	0.61
At1g30500	CCAAT-HAP2	0.08	0.60
At3g10760	GARP-G2-like	−0.58	−0.19
At1g54160	CCAAT-HAP2	0.71	−0.27
At3g12730	GARP-G2-like	0.35	−0.04
At1g72830	CCAAT-HAP2	−0.59	0.48
At3g13040	GARP-G2-like	−0.06	0.79
At2g34720	CCAAT-HAP2	0.51	0.77
At3g05690	CCAAT-HAP2	0.08	−0.43
At3g24120	GARP-G2-like	0.25	0.59
At1g17920	HB	−0.71	−0.02
At1g20693	HMG	0.09	0.62
At1g19700	HB	0.25	0.60
At1g20696	HMG	0.12	0.51
At1g20700	HB	0.24	0.40
At2g17560	HMG	0.01	0.52
At2g34450	HMG	0.28	0.23
At1g23380	HB	−0.88	1.05
At3g28730	HMG	0.80	0.27
At1g26960	HB	−0.45	1.02
At3g51880	HMG	0.10	0.53
At1g27050	HB	0.49	0.55
At4g11080	HMG	−0.35	0.29
At1g28420	HB	−0.31	0.62
At4g23800	HMG	0.21	−0.23
At1g30490	HB	0.09	−0.23
At4g35570	HMG	0.18	0.53
At5g23420	HMG	0.00	0.63
At1g46480	HB	−0.16	0.19
At5g56780	HRT-like	−0.12	0.31
At1g32330	HSF	0.43	0.53
At1g46264	HSF	−0.15	−0.27
At1g67970	HSF	1.40	2.26
At3g54340	MADS	−0.63	−1.55
At1g74250	HSF	0.02	0.25
At3g57230	MADS	0.04	0.67
At1g77570	HSF	0.15	1.19
At3g57390	MADS	−0.14	0.40
At2g26150	HSF	0.06	0.46
At3g58780	MADS	−0.13	0.61
At3g02990	HSF	0.97	1.27
At3g24520	HSF	2.41	2.91
At4g11250	MADS	3.71	2.40
At5g02320	MYB	0.03	0.74
At5g03780	MYB	−0.17	0.43
At1g73410	MYB	0.81	−0.27
At5g04110	MYB	0.04	0.24
At1g74080	MYB	−1.03	2.02
At5g06100	MYB	0.33	0.38
At1g74430	MYB	−0.15	1.02
At5g06110	MYB	0.30	0.61
At1g74650	MYB	−0.10	0.98
At5g07690	MYB	−0.55	0.45
At1g79180	MYB	0.43	1.73
At5g07700	MYB	−0.27	0.92
At2g02820	MYB	−0.09	0.79
At5g10280	MYB	0.85	0.40
At2g03470	MYB	−0.26	0.94
At5g11510	MYB	0.34	0.01
At3g51910	HSF	0.45	0.79
At4g11880	MADS	−1.34	0.22
At4g18960	MADS	−0.27	−0.42
At4g11660	HSF	0.41	0.50
At4g22950	MADS	−0.32	−0.37
At4g13980	HSF	−0.12	−0.47
At4g24540	MADS	0.11	0.42
At4g17600	HSF	0.15	0.65
At4g36590	MADS	−1.41	1.10
At4g17750	HSF	0.42	0.38
At4g37940	MADS	−0.17	1.43
At4g18880	HSF	0.70	2.89
At5g10140	MADS	−1.26	0.83
At4g36990	HSF	0.06	1.02
At5g13790	MADS	−0.09	0.52
At5g03720	HSF	0.69	1.22
At5g15800	MADS	0.06	−2.27
At5g16820	HSF	0.53	0.95
At5g20240	MADS	−1.28	−3.08
At2g16720	MYB	0.32	1.27
At5g14750	MYB	−1.85	0.49
At2g23290	MYB	−1.90	−1.21
At5g15310	MYB	0.05	0.29
At5g16600	MYB	0.75	0.84
At5g16770	MYB	0.76	−0.23
At5g17800	MYB	−0.71	−0.65
At2g31180	MYB	−0.09	1.35
At5g18620	MYB	0.93	−1.05
At5g23000	MYB	−0.74	−2.44
At2g33610	MYB	−0.16	0.22
At5g23650	MYB	0.40	−0.73
At2g36890	MYB	−0.40	1.54
At5g26660	MYB	−0.40	1.47
At2g36960	MYB	0.12	0.37
At5g35550	MYB	−0.24	−0.93
At2g37630	MYB	1.40	0.21
At5g43840	HSF	1.08	1.36
At5g23260	MADS	0.00	2.43
At5g45710	HSF	0.21	0.73
At5g26630	MADS	−0.68	0.91
At5g62020	HSF	0.86	1.65
At1g08620	JUMONJI	0.44	0.43
At5g26870	MADS	−0.06	1.91
At1g30810	JUMONJI	0.82	0.40
At5g26950	MADS	1.01	−2.59
At5g27050	MADS	0.51	3.03
At5g27070	MADS	−0.01	1.39
At2g38950	JUMONJI	0.36	0.45
At3g20810	JUMONJI	1.48	0.30
At3g48430	JUMONJI	0.64	0.46
At5g27580	MADS	0.09	−0.58
At2g39880	MYB	−0.19	−0.40
At5g40330	MYB	0.07	0.38
At2g42150	MYB	0.15	1.82
At2g44430	MYB	0.23	0.22
At5g40360	MYB	1.31	0.44
At2g47190	MYB	0.30	2.58
At2g47210	MYB	0.52	0.58
At5g41020	MYB	0.10	0.26
At2g47460	MYB	−1.20	1.24
At5g45420	MYB	−0.21	0.71
At2g47620	MYB	−0.10	0.15
At5g47290	MYB	2.40	1.34
At3g01140	MYB	−0.14	0.93
At5g49330	MYB	0.40	0.53
At3g01530	MYB	1.43	1.13
At5g52260	MYB	−0.05	2.22
At3g05380	MYB	0.92	−0.13
At5g52600	MYB	−1.74	−1.43
At3g06490	MYB	−2.33	1.21
At5g54230	MYB	−0.96	−1.03
At5g04240	JUMONJI	−0.05	0.65
At5g46910	JUMONJI	0.88	1.37
At5g37415	MADS	−1.00	3.14
At5g63080	JUMONJI	−0.08	0.56
At1g01780	LIM	0.92	0.41
At1g10200	LIM	0.03	0.71
At2g39900	LIM	−0.17	0.21
At2g45800	LIM	0.19	0.84
At3g55770	LIM	−0.45	0.57
At5g48670	MADS	−0.47	0.54
At4g32551	LUG	0.14	0.39
At5g49420	MADS	0.51	0.07
At5g65070	MADS	−0.07	0.94
At5g51860	MADS	1.09	0.89
At3g08500	MYB	−0.72	−0.10
At5g55020	MYB	2.33	5.47
At3g09370	MYB	0.32	0.47
At3g10113	MYB	−0.54	−0.73
At3g11440	MYB	0.67	0.76
At5g60890	MYB	0.94	1.21
At3g11450	MYB	−0.14	1.07
At5g61420	MYB	−0.22	0.34
At3g12560	MYB	0.20	0.13
At5g62320	MYB	0.21	1.80
At5g62470	MYB	1.39	2.19
At3g12820	MYB	−0.50	−0.05
At5g65230	MYB	0.79	1.02
At3g13540	MYB	0.80	1.04
At5g67300	MYB	0.70	1.61
At1g18750	MADS	0.75	0.66
At1g22590	MADS	0.89	0.28
At5g60440	MADS	−0.25	0.00
At1g24260	MADS	−0.54	−0.31
At5g60910	MADS	0.69	0.02
At5g62165	MADS	−1.47	−1.31
At1g28450	MADS	−1.54	1.09
At5g65050	MADS	0.05	0.25
At1g28460	MADS	−0.17	1.43
At5g65060	MADS	0.51	0.20
At1g29960	MADS	−0.15	0.61
At5g65080	MADS	0.62	−0.97
At2g42680	MBF1	−0.05	0.84
At3g24500	MBF1	−0.19	0.35
At3g15320	MYB	−0.12	0.63
At1g01060	MYB-related	0.19	0.95
At3g18100	MYB	0.27	0.44
At1g01380	MYB-related	−0.22	0.91
At3g23250	MYB	2.02	4.29
At1g01520	MYB-related	1.62	1.67
At1g09770	MYB-related	0.41	0.38
At3g27220	MYB	−1.04	−0.13
At1g15720	MYB-related	0.34	0.39
At1g17460	MYB-related	0.71	0.61
At3g27810	MYB	0.79	1.76
At3g27920	MYB	0.15	0.47
At1g18330	MYB-related	−0.49	−0.63
At1g19000	MYB-related	−0.21	1.13
At3g28910	MYB	0.20	0.99
At1g49950	MYB-related	−0.08	0.73
At1g70000	MYB-related	−0.29	0.22
At1g71030	MYB-related	−0.27	0.77
At1g33070	MADS	0.19	1.12
At3g58680	MBF1	0.11	0.85
At1g47760	MADS	−0.02	0.30
At1g06180	MYB	0.39	0.55
At1g48150	MADS	−3.43	1.85
At1g06910	MYB	0.95	1.06
At1g54760	MADS	3.53	−1.70
At1g59810	MADS	−0.54	0.67
At1g08810	MYB	−0.22	0.30
At1g09540	MYB	2.23	1.43
At1g09710	MYB	0.83	−1.33
At1g13880	MYB	−0.17	0.99
At1g14350	MYB	0.09	0.63
At1g16490	MYB	−0.25	0.85
At1g17950	MYB	0.48	0.55
At3g46130	MYB	−0.32	−0.09
At1g72650	MYB-related	−0.27	0.56
At3g47600	MYB	0.27	0.89
At1g72740	MYB-related	0.41	0.92
At3g47680	MYB	0.34	0.06
At1g74840	MYB-related	0.11	0.96
At3g48920	MYB	2.56	2.79
At1g75250	MYB-related	0.57	0.81
At3g49690	MYB	0.14	0.48
At2g21650	MYB-related	0.06	0.47
At3g50060	MYB	0.62	1.24
At3g52250	MYB	0.16	0.70
At2g30420	MYB-related	0.31	−0.02
At2g38090	MYB-related	−0.26	0.52
At3g55730	MYB	−0.11	0.52
At2g46410	MYB-related	−0.19	0.79
At3g57980	MYB	0.10	0.08
At2g46830	MYB-related	0.09	0.65
At3g60460	MYB	2.21	−1.95
At3g09600	MYB-related	0.08	0.46
At3g61250	MYB	−0.69	−0.17
At1g18570	MYB	1.43	3.26
At1g69540	MADS	0.14	0.11
At1g18710	MYB	1.17	2.64
At1g71692	MADS	−0.65	1.13
At1g18960	MYB	0.33	2.42
At1g19510	MYB	0.08	0.54
At1g77080	MADS	−0.16	0.37
At1g21700	MYB	0.00	0.32
At1g22640	MYB	0.33	1.00
At2g03060	MADS	1.28	0.51
At1g26580	MYB	−0.08	0.26
At2g03710	MADS	−0.02	0.89
At2g14210	MADS	0.85	−0.08
At2g22540	MADS	−0.03	0.50
At3g10590	MYB-related	−1.36	0.27
At3g11280	MYB-related	−0.04	0.62
At4g01680	MYB	−0.95	0.94
At3g16350	MYB-related	1.18	0.64
At4g01980	MYB	1.15	−0.75
At3g24870	MYB-related	−0.34	0.16
At4g05100	MYB	1.03	2.63
At3g49850	MYB-related	−0.32	0.38
At4g09460	MYB	0.75	1.86
At4g01060	MYB-related	−0.77	0.32
At4g12350	MYB	1.00	0.22
At4g01280	MYB-related	0.45	0.96
At4g16420	MYB	0.12	0.48
At4g11400	MYB-related	0.16	0.67
At4g36570	MYB-related	−0.93	0.12
At4g17785	MYB	0.95	1.66
At4g39250	MYB-related	1.23	−0.59
At5g01200	MYB-related	−0.30	0.17
At1g48000	MYB	0.32	3.79
At1g49010	MYB	0.04	−0.22
At2g26880	MADS	1.64	0.81
At1g56650	MYB	−0.73	3.60
At2g28700	MADS	−1.04	1.44
At1g57560	MYB	0.14	1.31
At2g34440	MADS	0.35	0.09
At1g58220	MYB	0.97	0.51
At1g63910	MYB	0.06	−0.06
At2g42830	MADS	−2.35	−1.26
At1g66230	MYB	−0.64	0.17
At2g45650	MADS	1.77	−2.34
At2g45660	MADS	0.07	0.45
At1g66380	MYB	0.89	5.32
At3g02310	MADS	5.56	−3.02
At1g66390	MYB	0.04	−0.20
At4g21440	MYB	1.72	2.48
At5g02840	MYB-related	−0.12	0.57
At4g22680	MYB	0.57	1.29
At5g04760	MYB-related	0.64	1.18
At5g05790	MYB-related	−0.26	0.39
At4g26930	MYB	−1.50	2.20
At5g08520	MYB-related	0.58	−0.03
At4g28110	MYB	1.18	2.55
At5g17300	MYB-related	0.42	1.05
At4g32730	MYB	0.83	0.08
At5g37260	MYB-related	2.68	3.28
At4g34990	MYB	0.24	0.09
At5g52660	MYB-related	0.73	1.50
At4g37260	MYB	0.31	1.47
At5g53200	MYB-related	0.34	0.57
At5g56840	MYB-related	−0.33	0.62
At4g38620	MYB	0.41	0.47
At5g58900	MYB-related	0.09	0.41
At5g47370	NAC	0.73	0.99
At5g67580	MYB-related	−0.17	0.60
At1g01010	NAC	0.48	1.17
At5g53950	NAC	1.56	−1.00
At1g01720	NAC	2.88	2.70
At5g53980	NAC	0.30	−0.46
At5g56620	NAC	0.93	1.00
At1g02220	NAC	−0.30	0.77
At5g59340	HB	2.22	1.42
At5g61430	NAC	0.49	−0.05
At5g62380	NAC	0.14	0.82
At5g63790	NAC	1.41	3.14
At1g12260	NAC	9.20	0.96
At5g64060	NAC	−0.12	−0.11
At5g64530	NAC	0.08	1.20
At1g19790	SRS	−0.88	−0.17
At2g18120	SRS	0.43	0.73
At1g66600	WRKY	0.53	1.60
At2g21400	SRS	−0.27	1.28
At1g69310	WRKY	0.63	0.51
At3g54430	SRS	−0.14	0.26
At1g69810	WRKY	0.89	1.49
At4g36260	SRS	0.33	0.12
At5g12330	SRS	−2.00	0.13
At1g80840	WRKY	1.49	6.06
At5g33210	SRS	0.21	0.79
At2g03340	WRKY	0.12	0.13
At5g66350	SRS	−0.18	0.07
At2g04880	WRKY	0.04	0.72
At1g05690	TAZ	−0.02	0.84
At1g25580	NAC	0.08	0.68
At5g65310	NAC	−0.10	0.20
At5g66300	NAC	0.03	0.77
At1g28470	NAC	−0.82	0.54
At1g32510	NAC	0.43	0.42
At5g39690	NAM	1.18	1.50
At5g50820	NAM	0.53	0.50
At1g32870	NAC	0.38	0.81
At1g33060	NAC	0.16	0.31
At1g20640	NIN-like	0.52	−0.06
At1g34180	NAC	0.22	0.15
At1g64530	NIN-like	0.25	0.25
At1g34190	NAC	0.11	0.92
At1g74480	NIN-like	−2.56	1.54
At1g52880	NAC	−0.10	0.56
At1g76350	NIN-like	−0.30	0.48
At1g52890	NAC	2.82	3.69
At2g17150	NIN-like	0.82	−0.12
At4g37610	TAZ	0.17	2.27
At2g23320	WRKY	2.07	3.14
At5g63160	TAZ	2.00	2.10
At2g24570	WRKY	0.55	0.82
At5g67480	TAZ	−0.44	1.05
At2g25000	WRKY	−0.33	0.07
At1g30210	TCP	−0.05	0.41
At2g30250	WRKY	0.63	2.13
At1g35560	TCP	0.03	0.91
At2g30590	WRKY	−0.44	0.40
At1g53230	TCP	0.24	0.83
At1g58100	TCP	0.14	0.37
At2g37260	WRKY	−0.50	0.63
At1g67260	TCP	−2.70	−0.70
At2g38470	WRKY	1.50	5.33
At1g68800	TCP	−0.10	1.83
At2g40740	WRKY	0.70	−0.15
At1g69690	TCP	−0.80	0.28
At2g40750	WRKY	0.49	0.37
At1g72010	TCP	0.43	0.61
At2g44745	WRKY	−1.08	0.27
At2g31070	TCP	0.01	0.02
At1g54330	NAC	−0.82	0.42
At2g43500	NIN-like	0.60	1.64
At1g56010	NAC	−0.50	0.39
At2g43500	NIN-like	0.46	0.49
At3g59580	NIN-like	−0.41	0.14
At4g24020	NIN-like	0.79	0.45
At4g35270	NIN-like	0.54	0.54
At4g35590	NIN-like	0.71	0.81
At1g62700	NAC	0.10	1.52
At1g64105	NAC	−0.07	0.47
At1g65910	NAC	0.50	0.67
At4g27330	NZZ	1.25	−5.05
At2g37000	TCP	0.43	0.10
At2g46400	WRKY	1.44	4.82
At2g45680	TCP	−0.06	−0.07
At2g47260	WRKY	0.30	0.53
At3g02150	TCP	−0.14	1.23
At3g01080	WRKY	−0.10	1.38
At3g15030	TCP	−0.20	−0.06
At3g01970	WRKY	0.53	1.93
At3g04670	WRKY	−0.07	0.62
At3g27010	TCP	0.50	1.13
At3g56400	WRKY	0.86	1.42
At3g58710	WRKY	0.79	0.92
At3g47620	TCP	0.33	0.89
At4g18390	TCP	0.17	0.44
At4g01250	WRKY	2.23	2.87
At5g08070	TCP	0.09	0.22
At4g01720	WRKY	2.13	1.22
At5g08330	TCP	−0.52	−0.21
At4g04450	WRKY	0.96	−0.24
At5g23280	TCP	−0.67	0.09
At1g69490	NAC	2.94	4.59
At1g71930	NAC	0.51	0.16
At5g35770	Orphan (SAP)	−2.53	−1.58
At1g76420	NAC	−3.79	−0.14
At1g14410	PBF-2-like(Whirly)	0.60	0.20
At1g77450	NAC	2.40	2.71
At1g71260	PBF-2-like(Whirly)	0.05	0.22
At2g02740	PBF-2-like(Whirly)	0.00	0.35
At2g02450	NAC	0.98	1.08
At1g05380	PHD finger	0.18	0.22
At2g36720	PHD finger	0.04	0.40
At2g18060	NAC	0.14	−0.31
At3g14980	PHD finger	−0.05	0.09
At2g24430	NAC	2.76	2.38
At3g53680	PHD finger	0.29	0.79
At2g27300	NAC	−0.16	1.23
At4g14920	PHD finger	0.70	0.50
At2g33480	NAC	−1.81	0.06
At5g12400	PHD finger	0.91	0.58
At2g43000	NAC	0.92	1.67
At5g22260	PHD finger	4.59	0.75
At5g41030	TCP	0.84	1.27
At4g12020	WRKY	0.71	0.40
At5g51910	TCP	−0.29	0.09
At4g18170	WRKY	1.99	3.02
At5g60970	TCP	0.14	0.27
At1g13450	Trihelix	0.07	0.60
At4g23550	WRKY	−0.28	−0.03
At1g21200	Trihelix	−0.14	0.45
At4g23810	WRKY	3.77	4.73
At4g24240	WRKY	0.88	1.69
At1g31310	Trihelix	0.89	0.55
At1g33240	Trihelix	−0.65	−0.47
At4g26640	WRKY	1.45	−0.24
At1g54060	Trihelix	−0.26	0.43
At4g30935	WRKY	0.11	0.42
At1g76880	Trihelix	0.98	0.08
At4g31550	WRKY	2.07	2.38
At1g76890	Trihelix	−0.54	−0.10
At4g31800	WRKY	1.41	2.71
At2g33550	Trihelix	0.61	0.52
At4g39410	WRKY	−0.03	0.99
At5g35210	PHD finger	0.07	0.70
At3g01600	NAC	−0.70	1.83
At3g03200	NAC	−2.55	0.74
At5g58610	PHD finger	−1.39	−1.08
At3g04060	NAC	0.37	0.02
At3g04070	NAC	−0.45	0.76
At1g21000	PLATZ	0.65	1.67
At1g31040	PLATZ	−1.87	0.25
At3g04420	NAC	−0.17	0.64
At1g32700	PLATZ	−0.16	1.10
At1g43000	PLATZ	2.53	3.44
At3g10480	NAC	0.88	0.14
At1g76590	PLATZ	1.42	1.77
At3g10490	NAC	0.81	0.07
At2g27930	PLATZ	1.89	1.39
At3g10500	NAC	0.26	0.94
At3g60670	PLATZ	−0.09	0.31
At3g15170	NAC	0.95	−2.63
At4g17900	PLATZ	1.01	1.84
At2g35640	Trihelix	2.40	2.93
At5g01900	WRKY	1.78	5.60
At2g38250	Trihelix	0.84	0.37
At5g07100	WRKY	2.13	1.12
At2g44730	Trihelix	0.14	0.61
At3g01560	Trihelix	1.10	0.47
At5g22570	WRKY	0.59	1.16
At3g10040	Trihelix	−1.27	−0.35
At5g24110	WRKY	1.46	4.27
At3g11100	Trihelix	−0.20	0.44
At5g26170	WRKY	−0.25	2.65
At3g14180	Trihelix	0.07	0.81
At5g28650	WRKY	−0.01	−0.61
At3g19020	Trihelix	1.43	1.18
At3g24490	Trihelix	−0.22	0.42
At5g43290	WRKY	0.27	0.87
At3g24860	Trihelix	−0.07	0.35
At5g45050	WRKY	0.37	0.28
At3g25990	Trihelix	0.10	0.31
At5g45270	WRKY	0.76	0.96
At3g15500	NAC	2.51	3.62
At5g46710	PLATZ	1.85	2.23
At3g15510	NAC	0.51	0.57
At3g17730	NAC	0.40	1.19
At4g02020	Polycomb Group (PcG)	0.28	0.50
At3g29035	NAC	−0.40	1.40
At4g16845	Polycomb Group (PcG)	−0.08	0.09
At5g51230	Polycomb Group (PcG)	0.26	0.22
At3g27700	RRM-containing	0.52	0.78
At4g01540	NAC	0.33	2.07
At3g47120	RRM-containing	0.11	0.39
At4g27410	NAC	1.65	3.18
At2g37120	S1Fa-like	−1.00	0.63
At4g28500	NAC	−0.19	−0.07
At3g53370	S1Fa-like	0.11	0.66
At4g28530	NAC	−0.38	0.93
At1g02065	SBP	0.44	−0.72
At3g54390	Trihelix	−0.39	−0.11
At5g46350	WRKY	0.26	1.24
At3g58630	Trihelix	0.00	0.16
At5g49520	WRKY	1.74	2.73
At4g17050	Trihelix	−0.24	0.41
At5g52830	WRKY	0.93	1.22
At4g31270	Trihelix	−0.37	0.59
At5g56270	WRKY	−0.06	−0.03
At5g01380	Trihelix	−0.10	2.40
At5g64810	WRKY	0.78	2.26
At5g03680	Trihelix	−0.41	0.87
At1g14440	ZF-HD	−0.18	−0.17
At5g05550	Trihelix	−0.01	0.38
At1g14687	ZF-HD	0.06	0.36
At5g14540	Trihelix	3.11	0.00
At5g28300	Trihelix	−0.18	−0.58
At1g74660	ZF-HD	−0.26	0.00
At5g38560	Trihelix	0.48	0.69
At1g75240	ZF-HD	−0.45	−0.13
At5g47660	Trihelix	0.02	0.15
At2g02540	ZF-HD	−0.10	0.60
At5g63430	Trihelix	0.35	0.47
At2g18350	ZF-HD	0.34	0.27
At4g35580	NAC	0.59	0.36
At1g20980	SBP	0.39	0.60
At4g36160	NAC	0.08	1.12
At1g27360	SBP	0.91	0.64
At5g04400	NAC	1.31	1.79
At1g27370	SBP	0.56	0.01
At5g04410	NAC	0.21	0.52
At1g53160	SBP	−0.51	−0.46
At5g07680	NAC	−0.71	−0.44
At1g69170	SBP	−0.24	0.19
At5g08790	NAC	1.56	1.81
At2g33810	SBP	−0.01	0.12
At5g09330	NAC	0.76	0.34
At2g42200	SBP	−0.45	−0.37
At5g13180	NAC	−0.45	0.17
At2g47070	SBP	0.22	0.60
At5g14000	NAC	−0.57	−0.15
At3g15270	SBP	−0.51	−0.35
At3g57920	SBP	0.27	−0.36
At5g17260	NAC	0.22	0.79
At3g60030	SBP	0.22	0.30
At5g18270	NAC	0.31	0.84
At5g18830	SBP	0.04	0.26
At1g16070	TUB	−0.19	−0.25
At3g28920	ZF-HD	0.06	0.23
At1g25280	TUB	0.80	0.36
At3g50890	ZF-HD	−0.47	−0.58
At4g24660	ZF-HD	−0.36	−0.01
At1g47270	TUB	0.35	0.68
At5g15210	ZF-HD	−0.12	0.01
At5g39760	ZF-HD	0.84	0.07
At5g42780	ZF-HD	−0.17	−0.33
At1g76900	TUB	1.50	0.46
At2g18280	TUB	0.19	0.55
At5g65410	ZF-HD	1.11	0.15
At2g47900	TUB	1.25	0.11
At1g17380	ZIM	1.91	3.30
At3g06380	TUB	−0.02	0.64
At1g19180	ZIM	2.60	4.54
At5g18680	TUB	−0.10	0.44
At1g30135	ZIM	−0.37	2.56
At1g48500	ZIM	1.18	0.17
At5g18300	NAC	−0.83	−0.13
At5g43270	SBP	−0.64	−0.77
At5g22290	NAC	0.28	1.92
At5g50570	SBP	0.26	1.42
At5g22380	NAC	3.76	4.76
At5g50670	SBP	0.30	0.91
At5g24590	NAC	1.15	1.53
At1g05830	SET-domain	0.18	−0.06
At5g39610	NAC	0.19	2.46
At2g31650	SET-domain	0.27	0.57
At5g39820	NAC	1.91	0.05
At4g27910	SET-domain	0.29	0.60
At5g41410	NAC	0.08	−0.03
At4g30860	SET-domain	−0.13	0.71
At5g09790	SET-domain	0.48	0.50
At5g24330	SET-domain	−0.05	0.27
At5g46590	NAC	0.08	1.50
At5g53430	SET-domain	0.30	0.17
At4g28190	ULT	−0.26	0.02
At1g70700	ZIM	0.14	1.73
At1g28520	VOZ	0.32	0.75
At1g72450	ZIM	−0.27	1.16
At2g42400	VOZ	0.24	0.44
At1g74950	ZIM	0.27	0.93
At2g34600	ZIM	−1.53	3.07
At3g17860	ZIM	0.10	1.25
At1g29280	WRKY	−0.40	2.45
At3g43440	ZIM	0.15	0.16
At1g29860	WRKY	−0.16	1.03
At4g14713	ZIM	0.94	−0.11
At4g14720	ZIM	−0.01	0.21
At4g32570	ZIM	0.50	0.50
At1g62300	WRKY	2.04	2.08
At5g13220	ZIM	0.10	1.75
At5g20900	ZIM	0.22	1.13

Values are means of two biological replicates.
